# Temporal consistency and ecological validity of personality structure in common marmosets (*Callithrix jacchus*): A unifying field and laboratory approach

**DOI:** 10.1002/ajp.23229

**Published:** 2021-01-19

**Authors:** Vedrana Šlipogor, Jorg J. M. Massen, Nicola Schiel, Antonio Souto, Thomas Bugnyar

**Affiliations:** ^1^ Department of Behavioral and Cognitive Biology University of Vienna Vienna Austria; ^2^ Department of Biology Federal Rural University of Pernambuco Recife Brazil; ^3^ Department of Zoology Federal University of Pernambuco Recife Brazil; ^4^ Animal Ecology Group, Institute of Environmental Biology, Department of Biology Utrecht University Utrecht The Netherlands

**Keywords:** long‐term consistency, New World monkeys, nonhuman primate personality, personality assessment, personality comparison

## Abstract

Personality in animals has been extensively researched in recent decades. Temporal consistency of behaviors is almost always part of the personality definition and is usually explored in several different testing sessions or observation periods. However, it is still unclear whether the obtained personality constructs are stable across several years, which might be especially important for long‐living animals, such as primates. Further, little is known on whether the personality structures obtained in the laboratory reflect the structures obtained under ecologically relevant conditions in the wild. Therefore, we conducted a battery of personality tests on common marmosets (*Callithrix jacchus*) (*N* = 27), compared it with a test battery conducted 4 years beforehand on a subset of animals in captivity (*N* = 13) and ran an adapted version under field conditions at Baracuhy Biological Field Station, Brazil (*N* = 18). Under captive conditions, we found a remarkably similar personality structure across 4 testing years. Further, we found high long‐term temporal consistency in the first two personality components, Boldness and Exploration; however, monkeys that changed their social (i.e., breeding) status between the two testing periods showed a significant increase in Boldness scores. Under field conditions, we found a somewhat similar personality structure as compared to the laboratory, which to some extent corroborates ecological validity of our personality test design. Nevertheless, whether the structure in the wild is suppressed or expanded in comparison to captivity, and in which way the social setting influences personality structure, should be further explored. Taken together, our results contribute to the discussion about the reliability and ecological validity of personality structures in nonhuman primates.

## Research Highlights

- Personality structure was similar and Boldness and Exploration traits were stable across 4 years in captive marmosets.
- Boldness increased in individuals that changed social status.
- Wild personality structure corresponded to the captive structure.

## Graphical abstract

Captive common marmosets that changed social status in the group, increased their Boldness scores.

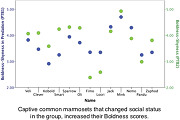

## 1 INTRODUCTION

Animal personality has been extensively researched in recent decades and described in many species, from invertebrates to humans (Freeman & Gosling, 2010; Gartner & Weiss, 2013; Koski, 2014; Kralj‐Fišer & Schuett, 2014; Réale et al., 2007; Weiss, 2018; Wilson et al., 2019). Personality has been linked with fitness (Adriaenssens & Johnsson, 2013; Seyfarth et al., 2012; Smith & Blumstein, 2008), development (Delval et al., 2020; Polverino et al., 2016; Stamps & Groothuis, 2010; von Borell et al., 2019; Wuerz & Krüger, 2015), longevity (Altschul et al., 2018; Careau et al., 2010), subjective well‐being (Gartner et al., 2016; Robinson et al., 2016; Weiss et al., 2009), choice of social partners (Ebenau et al., 2019; Massen & Koski, 2014; Verspeek et al., 2019), social information use (Carter et al., 2013; Carter et al., 2014a; Kurvers et al., 2010) and increasingly also cognition (Altschul et al., 2017; Dougherty & Guillette, 2018; Mazza et al., 2018), and genetics (Staes et al., 2019; Wilson et al., 2017).

Nonhuman primate personality has usually been studied in captivity (e.g., Capitanio 1999, 2004; Koski, 2011; Masilkova et al., 2018; Šlipogor et al., 2016, 2020; Stevenson‐Hinde & Zunz, 1978; Stevenson‐Hinde et al., 1980a, 1980b; Uher & Visalberghi, 2016; Uher et al., 2008; Uher et al., 2013; Wilson et al., 2018), and, to a somewhat lesser extent, also in the wild (e.g., Fernández‐Bolaños et al., 2020; Carter et al., 2014b; Dammhahn & Almeling, 2012; Ebenau et al., 2020; Eckardt et al., 2015; Garai et al., 2016; Konečná et al., 2008; Manson & Perry, 2013). Personality trait correlations are likely maintained by mechanisms that may either constrain behavioral changes (i.e., “constraint hypothesis”; Bell, 2005; Lande, 1979) or particular traits may be selected for in some distinct environments (i.e., “adaptive hypothesis”; Bell, 2005; Lande & Arnold, 1983). It has been argued that personality structure as described in captivity could potentially show less behavioral variability than under field conditions and, in fact, be an adaptation to the particular environment (McDougall et al., 2006), as selection pressures largely differ between these distinct environments (Adriaenssens & Johnsson, 2009; Smith & Blumstein, 2008). Thus, to investigate the evolutionary and ecological significance of personality structure, results from captivity should be compared with those obtained under natural settings (Adriaenssens & Johnsson, 2013; Herborn et al., 2010), but such studies are still quite rare (e.g., Fisher et al., 2015). Assessing primate personality in the wild, usually by means of behavioral observations, behavioral testing, or questionnaires is not very commonly done, most likely due to logistical reasons (Tkaczynski et al., 2019). Using behavioral testing in the wild has some further challenges, such as testing individuals repeatedly, the inconsistency of solitary and social testing situations, and ensuring the “novelty” aspect of the testing set‐up to all tested individuals; however, if implemented successfully (as in e.g., Carter et al., 2012a; 2012b; Dammhahn & Almeling, 2012; Dammhahn, 2012), such studies can provide insight into a wide range of otherwise rare behaviors, like animals' natural reactions to predators.

In both captive and natural settings, animal personality has been defined in a variety of ways, from “consistent interindividual behavioral differences that are stable throughout time and/or across different contexts” (Réale et al., 2007) to “stable individual differences in behaviors, emotions, and thinking” (Pervin & John, 1997). Regardless of the discipline, the centerpiece of all definitions and main criterion of personality is the consistency or stability of measured behaviors, and this is usually explored by conducting several testing or observation sessions at different points in time (Guenther et al., 2014). A given behavior is considered temporally consistent if, in statistical terms, significant repeatability is found across several testing sessions (Bell et al., 2009). Guidelines on an “optimal” between‐testing period length are, however, somewhat vague and largely depend on the life cycle of the species in question. Studies using animals with short life cycles, for example, invertebrates, report a relatively short period between two repeated tests of several hours to days (Fisher et al., 2015; Schuett et al., 2011), while studies on most mammals and birds make longer gaps between testing, that is, several weeks or months (Miller et al., 2015; Webb et al., 2017; Wuerz & Krüger, 2015; Herde & Eccard, 2013). However, it is still unclear whether the obtained personality constructs are stable across longer time periods (e.g., several years), which might have special importance for long‐living species, such as primates (but see e.g., Dutton, 2008; Stevenson‐Hinde et al., 1980a; Weiss et al., 2017; Zablocki‐Thomas et al., 2018).

Common marmosets (*Callithrix jacchus*) are highly social cooperatively breeding New World primates that live in cohesive family groups in a variety of different habitats, from the Atlantic rain forest to the semiarid area of shrub forests (Garber et al., 2019) and have been studied in a variety of socio‐cognitive questions (Schiel & Souto, 2017). In recent years, these monkeys have become the focus of personality studies under lab conditions: they display consistent interindividual differences when assessed in a battery of experiments (Díaz et al., 2020; Koski & Burkart, 2015; Šlipogor et al., 2016; Tomassetti et al., 2019), observations (Martin et al., 2019; Masilkova et al., 2020; Šlipogor et al., 2020), questionnaires (Inoue‐Murayama et al., 2018; Koski et al., 2017; Weiss et al., 2020), and by using a combination of several different personality assessment methods (Iwanicki & Lehmann, 2015; Šlipogor et al., 2020). However, little is known about long‐term consistency of their personality structure, and no study to this date assessed the personality of wild common marmoset populations.

In this study, we addressed the questions of (i) temporal consistency of common marmoset personality structure and individual monkeys' personality scores across 4 years and (ii) whether these personality structures from captivity reflect the structures obtained in the wild. We first conducted a battery of personality tests on captive animals that were tested 4 years beforehand in a personality test battery (Šlipogor et al., 2016) and predicted that the overall personality structure should be highly similar as both the individual testing procedure and the maintenance of the animals were kept the same in these two studies. We then conducted this battery of tests adapted for natural conditions in Brazil. We predicted that the personality structure in the natural setting should overall correspond to the structure in captivity as maintenance of these monkeys in captivity mimics the natural conditions, and captive common marmosets' behavioral repertoire is largely the same as in the wild (Stevenson & Poole, 1976; Stevenson, 1978). However, partly due to testing in family groups, in contrast to the individual tests in captivity, the personality structure of the wild population might either be (a) enhanced, and entail more personality traits, as it has been suggested that wild individuals might show a larger spectrum of behaviors and have a higher between‐individual variance than the captive individuals (McDougall et al., 2006), or (b) suppressed, and entail fewer personality traits, due to within‐group social dynamics that might restrict the full range of shown behaviors (Webster & Ward, 2011).

## 2 METHODS

### 2.1 Ethics statement

The study in the captive population was approved by the Animal Ethics and Experimentation Board, Faculty of Life Sciences, University of Vienna (license number 2015‐013) and adhered to the legal requirements of Austria. The study in the wild population adhered to the legal requirements of Brazilian laws governing wild animal research (SISBio no. 46770‐2) and was approved by the Ethics Committee for Animal Use of the Federal Rural University of Pernambuco (CEUA no. 144/2014). This study also complied with the Code for Best Practices in Field Primatology. Both studies adhered to the American Society of Primatologists' Principles for the ethical treatment of primates. All applicable international, national, and institutional guidelines for the care and use of animals were followed.

### 2.2 Study sites and populations

We studied the marmoset colony of the Department of Behavioral and Cognitive Biology, University of Vienna, Vienna, Austria (UVI Austria). Monkeys were housed in two keeping rooms in their family groups in indoor–outdoor enclosures (per group about 5 m × 2.5 m × 2.5 m), that were visually isolated from each other, but in acoustic and olfactory contact. The enclosures were equipped with branches, bamboos, wooden boards, baskets, tunnels, hammocks, towels, and toys, and the floors of indoor enclosures were covered with coniferous pellets. Both keeping rooms had windows for natural light, and additional artificial lights were available (with a day:night cycle set to 12:12 h), as well as infrared lamps placed above the enclosures, to improve the well‐being of subjects. Every enclosure was inter‐connected via a passageway tunnel system and further linked to the smaller experimental cages (152 cm × 42 cm × 110 cm). The room temperature was maintained between 21°C and 29°C and the humidity was kept between 30% and 60%. All monkeys had *ad libitum* access to water. The varied and well‐balanced food diet included vitamin‐ and mineral‐rich New World monkey pellets, fruits, vegetables, eggs, nuts, insects, marmoset gum and jelly, and was served twice a day during the testing period. Additionally, monkeys regularly obtained foraging boxes with insects, granola, tea or frozen fruit pulp as enrichment. The housing conditions were in accordance with the Austrian legislation and the European Association of Zoos and Aquaria (EAZA) husbandry guidelines for Callitrichidae. We tested 27 monkeys from five different family groups, 10 females and 17 males (0.5–14 years; see Table S1), between February and July 2016.

The study on wild marmosets was conducted at the Baracuhy Biological Field Station, located at Fazenda Marimbondo (7°31′42″S, 36°17′50″W), in the municipality of Cabaceiras, Paraíba, Brazil (BBFS Brazil). The location is considered a hot semiarid type, has limited rainfall, shallow and rocky soils, and low vegetation consisting of arboreal shrubs and scattered trees (De la Fuente et al., 2014). During the data collection time, the mean temperatures ranged from 18.5°C to 35.7°C according to the Brazilian National Institute for Meteorology (INMET), Cabaceiras station. Most marmosets of the area were well habituated to the presence of humans from previous studies (e.g., Abreu et al., 2016, 2019; Caselli et al., 2018; De la Fuente et al. 2014, 2019). In particular, three groups (Casa, Coqueiro, and Vacas) were already habituated to humans, whereas two groups (Star Wars and Azul) were newly habituated for this study. The activity of the groups started with the dawn and ended approximately with the dusk, with a resting period around midday (De la Fuente et al., 2014). We studied 18 monkeys living in five different family groups, 8 females and 10 males, between March and May 2017. The exact ages of monkeys were unknown, but we assigned age classes following Yamamoto (1993) and Schiel and Huber (2006), dividing them into juveniles (approx. 5–10 months, *N* = 5), subadults (approx. 11–15 months, *N* = 2), and adults (above 16 months, *N* = 11) (see Table S1). The individuals were identified using sex, age, social status within the group, as well as natural markings (i.e., facial and bodily features).

### 2.3 Personality tests

#### 2.3.1 Habituation and experimental procedure in captive population (UVI Austria)

In Vienna, we established a personality test battery in 2012 (Personality Test Battery 1, PTB1), using five different tests and their controls, in two testing sessions. The second testing session was conducted 2‐weeks apart from the first testing session, to assess the short‐term temporal and contextual consistency in PTB1 (see details in Šlipogor et al., 2016). We then repeated the testing battery 4 years afterwards, that is, in 2016, in the Personality Test Battery 2 (PTB2). Again, we tested subjects in these five tests, in two sessions, with a 2‐week gap between the sessions, to assess their short‐term temporal and contextual consistency in PTB2. The five tests used in both PTB1 and PTB2 were (i) General Activity (GA), measuring the baseline of subjects' activity while being exposed to an experimental situation, (ii) Novel Object (NO), exposing subjects to a novel object (i.e., a plastic multicolored round toy in the first test session, a plastic multicolored rattle‐shaped toy in the second test session, in PTB2), (iii) Novel Food (NF), exposing monkeys to a novel food placed on a ceramic plate (i.e., a piece of star fruit in the first test session, a piece of jackfruit in the second test session, in PTB2), (iv) Foraging Under Risk (FUR), where we simultaneously exposed monkeys to highly desirable food rewards (i.e., five meal worms) and a frightening stimulus (i.e., lychee fruit with skin, as it was established previously that monkeys emit a mobbing, predator‐like response to a lychee fruit, most likely due to its visual resemblance to snake skin; see Šlipogor et al., 2016), and (v) Predator (P), exposing subjects to a predator model (i.e., plastic toy snake) hidden in leaves.

All tests were conducted in a small experimental cage (see the experimental set‐up, Figure S1). Before the first test session of PTB2 began, the subjects received a 2‐week habituation phase, in daily sessions of 30 min. In this phase, the subjects had access to the experimental cage filled with food rewards, the passageway system, the experimental routine and the experimenter (VŠ), first in family groups and then individually. Each test started once the entrance of the experimental cage was opened and it lasted for 300 s. The tunnels closest to the entrance were mostly opaque to prevent subjects from seeing the experimental set‐up before the start of the tests and for giving them a hiding place (especially during P and FUR tests, i.e., tests with predator models). The experimental set‐up was placed on a plastic plate in the furthest point of the experimental cage (set diagonally to the entrance door). The plastic plate was exchanged and the cage was thoroughly washed with a vinegar‐water solution between two subjects, to avoid any possible olfactory interference. For the purpose of analysis, we virtually divided the experimental cage into four compartments, with an additional fifth compartment consisting only of the tunnel before the entrance door. Thus, the compartment containing the plate represented “proximity” to the set‐up, whereas the one furthest away from it, together with the tunnel before the entrance door (i.e., fourth and fifth compartments), represented “distance” from the set‐up. To minimize the possibility of habituation, stimuli in novelty tasks (NF and NO) were used only once per session. All subjects were tested individually in one test per testing day, with a 72‐h break between two tests. In the 2‐week break between the two sessions, the monkeys did not participate in any other experiment in the laboratory. The order of subjects within each testing day was randomized. While the GA test was always conducted as the first test for all subjects, the subsequent starting tests for each subject were randomized (NO, NF, FUR, or P), but the order of the tests was kept the same (NO–NF–FUR–P). For example, a subject assigned with an NF as a starting test had a test order of GA–NF–FUR–P–NO. All tests were conducted in the morning hours (9:00–12:00 am). Before the tests, the subjects received their breakfast which consisted of New World monkey pellets and after the tests, they received their full lunch. Water was available *ad libitum*.

We recorded subjects' behavior in tests from two different angles using two video cameras (Canon Legria HF G25). One camera, placed on a tripod, filmed the close‐up of the experimental set‐up, while the other camera was handled by VŠ, and focused on the subject. We synchronized and further edited the two‐angled videos into a single video, using a video editing software (CyberLink Power Director, version 15). We analyzed the videos using Solomon Coder beta v. 17.03.22 (Péter, 2017). We measured the same behavioral variables in all tests (Table 1). Additionally, in NF we measured *Ingestion‐Related Behavior* ^F^, and in FUR we measured *Ingestion‐Related Behavior* ^F^, *Inspection Lychee* ^F^, and *Route*.

**Table 1:** Behavioral variables in captivity (UVI Austria) coded, their descriptions and tests in which they were measured

Behavioral variable	Description	Tests				
Enter^L^	Latency (i.e., time it takes the subject) to enter into the experimental cage, with full body without tail.	GA	NO	NF	FUR	P
Body^L^	Latency (i.e., time it takes the subject) to be within one body length of the stimulus/object/food.	GA	NO	NF	FUR	P
Touch^L^	Latency (i.e., time it takes the subject) to touch the stimulus/object/food.	GA	NO	NF	FUR	P
Vigilance Calls^F^	Number of times the subject emits following calls: tsik, rapid tsik, tsik‐egg, egg, cough, chatter, loud shrill.	GA	NO	NF	FUR	P
Contact Calls^F^	Number of times the subject emits following calls: twitter, phee, loud phee, see, trill.	GA	NO	NF	FUR	P
Food Calls^F^	Number of times the subject emits following calls: chirp.	GA	NO	NF	FUR	P
Self‐grooming^F^	Number of times the subject grooms itself (i.e., goes with hands or mouth through own fur).	GA	NO	NF	FUR	P
Stress Behavior^F^	Number of times the subject shows behaviors indicative of stress, e.g., scent marks parts of the cage or the tunnel system, scratches itself, has pilo‐erected fur, defecates, urinates or manipulates the cage in a destructive manner.	GA	NO	NF	FUR	P
Inspection Cage^F^	Number of time the subject licks/smells the wire mesh, floor or other parts of the experimental cage.	GA	NO	NF	FUR	P
Compartment Alternations^F^	Number of times the subject changes virtual compartments, with full body, without tail.	GA	NO	NF	FUR	P
Locomotion^D^	The duration of time that the subject spends walking, running, climbing, or jumping, with or without holding/manipulating/eating, etc. stimulus/object/food (any movement in the experimental cage).	GA	NO	NF	FUR	P
Proximity^D^	The duration of time that the subject is in closest proximity to the experimental set‐up (lower quarter of the experimental cage, i.e., virtual compartment placed diagonally to the subject's entrance point).	GA	NO	NF	FUR	P
Ground^D^	The duration of time that the subject is on the ground (lower part of the cage, i.e., in the first or second virtual compartment.	GA	NO	NF	FUR	P
Distance^D^	The duration of time that the subject is furthest away from the experimental set‐up (upper quarter of the experimental cage including the tunnel before the entrance door to the experimental cage, i.e., virtual compartment placed diagonally to the experimental set‐up).	GA	NO	NF	FUR	P
Focus^D^	Duration of time that the subject is looking at the stimulus/object/food (i.e., head turned to the stimulus/object/food).	GA	NO	NF	FUR	P
Manipulation^D^	Duration of time that the subject actively manually or orally manipulates (i.e., touches, bites, licks or scratches), smells and/or eats/tries to eat the stimulus/object/food.	GA	NO	NF	FUR	P
Ingestion‐Related Behavior^F^	Number of times the subjects eats/drinks, licks lips and is actively looking for food rewards in the sawdust.			NF	FUR	
Inspection Lychee^F^	Number of times subject actively manually or orally manipulates lychee (i.e., touches, bites, licks or scratches), smells and/or eats/tries to eat lychee.				FUR	
Route	Route an individual takes to get from the entrance to the food rewards (0 = direct, 1 = indirect, and 2 = no route)				FUR	

*Note*: Frequencies are noted with the letter “F” in superscript, durations with the letter “D” in superscript, and latencies with the letter “L” in superscript.

Abbreviations: FUR, Foraging Under Risk; GA, General Activity; NO, Novel Object; NF, Novel Food; P, Predator.

We calculated personality structure separately for PTB1 (see Table S11) and PTB2. Finally, we compared the obtained personality components in PTB1 and PTB2 for long‐term temporal consistency (i.e., from 2012 to 2016).

#### 2.3.2 Habituation and experimental procedure in wild population (BBFS Brazil)

All testing and training sessions were conducted on two wooden platforms (50 cm × 55 cm) that were positioned in the most often used area in the family groups' home range, as determined in previous studies or during the habituation. The platforms were approximately 100 cm high, with a 100 cm distance from each other that enabled subjects to easily move between them. Additionally, each group had either a tree trunk or a tree branch attached, or very close to the platforms, for easier reachability from adjacent vegetation. All tests were done on these two platforms to ensure that the set‐up resembles the set‐up in the lab (see Figure S2).

We followed the design of laboratory tests, but with some adjustments to the field conditions: notably, we used a Startle Response (SR) test instead of the FUR test, to test for the personality trait Boldness–Shyness. The test was designed to allow a simultaneous exposure to highly desirable food pieces and a remotely controlled stimulus that was initiated once > 50% of the family group members ate from the platform with bananas, to “startle” the subjects.

Before conducting tests, VŠ and a field assistant habituated each group to their presence with positive reinforcement techniques twice daily for a period of two and a half weeks. Before the start of testing, three training sessions were conducted in which the monkeys were provided with banana pieces on both platforms. One additional training session was conducted in the afternoon preceding the first testing day with groups that had a longer gap between the training and testing sessions. Each family group was then tested on five consecutive days, with one test per day and a 24‐h break between two different tests. The order of tests was always the same (GA–NO–NF–SR–P), however, apart from GA, each group had a different starting test which was randomly assigned (NO, NF, SR or P). To minimize the risk of subjects getting habituated to novelty, stimuli in novelty tasks (NO and NF) were new in both testing sessions. In NO tests, we used the same two objects as in captivity; namely, a round multicolored toy in the first, and a rattle‐shaped multicolored toy in the second testing session. In NF tests, we used pieces of grapes in the first testing session and pieces of guava fruit in the second testing session. At maximum, two family groups were tested on the same day. Every test was conducted in both testing sessions and with a 2‐week break between the sessions, to test for short‐term temporal repeatability.

To ensure that all monkeys came to the platforms, we placed approximately 60 g of banana (i.e., one half of banana), cut into pieces, onto one platform, and hid them with a container and a piece of grey cloth. We placed the experimental set‐up on the other platform (novel object, novel food, remote‐controlled toy, plastic toy snake hidden with leaves and branches) and covered it with a container and a piece of grey cloth. First, we uncovered the platform containing bananas. We waited until approximately 50% of the group members came 1 m away from the platform, and then uncovered the other platform with an experimental set‐up, what marked the official start of the test. The test ended after 300 s. Afterward we covered the platforms again and cleaned them. For the purpose of video analysis, we virtually divided the whole experimental setting into different compartments. The compartments containing the experimental set‐up together with the tree or the branch either above the set‐up or below it, was considered “proximity” (i.e., area of about 1 m around the experimental set‐up), whereas the platform containing the banana pieces and the further tree or branch below the platform or above it was considered “middle” (i.e., area of about 1 m around the platform with banana pieces). Everything outside of these areas was considered “distance". All tests were conducted between 5:30 and 7:30 am. We filmed the experimental set‐up with an HD camcorder Canon Legria HF G25, and noted behaviors, positions and social interactions of each individual during the test (Table 2). We measured the same behavioral variables in all tests, but in two tests we measured two additional variables, namely *Nb Eaten Target* ^F^ in NF and *Return* ^L^ in SR. The camera was placed on a tripod approximately 3 m away from the platforms (focusing on both platforms and, when necessary, zoomed into the experimental set‐up).

**Table 2:** Behavioral variables in wild (BBFS Brazil) coded, their descriptions and tests in which they were measured

Behavioral variable	Description	Tests				
Platform^L^	Latency (i.e., time it takes the subject) to come to one of the two experimental platforms, with one or more hands/legs. The time is measured from the moment the platform with bananas is revealed. The maximum latency is 300 s plus the time between the initial reveal of the banana platform and the reveal of stimulus.	GA	NO	NF	SR	P
Body^L^	Latency (i.e., time it takes the subject) to be within one body length of the stimulus/object/food.	GA	NO	NF	SR	P
Touch^L^	Latency (i.e., time it takes the subject) to touch the stimulus/object/food.	GA	NO	NF	SR	P
Return^L^	In SR test, latency (i.e., time it takes the subject) to return to the platform with bananas after the remotely‐controlled toy has moved.				SR	
Vigilance Calls^F^	Number of times the subject emits following calls: tsik, tsik‐egg, egg, cough, chatter.	GA	NO	NF	SR	P
Contact Calls^F^	Number of times the subject emits following calls: twitter, phee, loud phee, see, trill.	GA	NO	NF	SR	P
Food Calls^F^	Number of times the subject emits following calls: chirp.	GA	NO	NF	SR	P
SUM Calls^F^	Number of times the subject emits vigilance, contact, and food calls.	GA	NO	NF	SR	P
Self‐grooming^F^	Number of times the subject grooms itself (i.e., goes with hands or mouth through own fur).	GA	NO	NF	SR	P
Stress Behavior^F^	Number of times the subject shows behaviors indicative of stress, e.g., scratches or shakes itself, has pilo‐erected fur, defecates, urinates, gives an alarm, startles, scent marks, gnaws or manipulates surface in a destructive manner.	GA	NO	NF	SR	P
Nb Eaten Target^F^	Number of food items that a subject eats during the test.			NF		
Compartment Alternations^F^	Number of times the subject changes virtual compartments, with full body, without tail.	GA	NO	NF	SR	P
Locomotion^D^	The duration of time that the subject spends walking, running, climbing, or jumping, with or without holding/manipulating/eating, etc. stimulus/object/food (any movement).	GA	NO	NF	SR	P
Proximity^D^	The duration of time that the subject is in closest proximity to the experimental set‐up (in the first compartment, i.e., on the stimulus platform or in the third compartment i.e., approximately 1 m above and below the stimulus platform).	GA	NO	NF	SR	P
Platform^D^	The duration of time that the subject is on the banana or stimulus platforms.	GA	NO	NF	SR	P
Distance^D^	The duration of time that the subject is furthest away from the experimental set‐up (approximately 2‐3 meters away from the stimulus; i.e., not close to first, second, third, or fourth compartment).	GA	NO	NF	SR	P
Focus^D^	Duration of time that the subject is looking at the stimulus/object/food (i.e., head turned to the stimulus/object/food).	GA	NO	NF	SR	P
Manipulation^D^	Duration of time that the subject actively manually or orally manipulates (i.e., touches, bites, licks, or scratches), smells and/or eats/tries to eat the stimulus/object/food.	GA	NO	NF	SR	P
Sociopositive Initiate^F^	Number of grooming events (i.e., using hand or mouth to pick through hair or skin of body parts of another individual) or playing events that the focal animal initiated to others.	GA	NO	NF	SR	P
Socionegative Initiate^F^	Number of chatters, threats, bites, conflicts, body attacks, chases that the focal animal initiated to others.	GA	NO	NF	SR	P

*Note*: Frequencies are noted with the letter “F” in superscript, durations with the letter “D” in superscript, and latencies with the letter “L” in superscript.

Abbreviations: FUR, Foraging Under Risk; GA, General Activity; NO, Novel Object; NF, Novel Food; P, Predator.

### 2.4 Data analysis

We analyzed the data in SPSS Statistics v. 23 (IBM). To minimize observer bias, approximately 10% of videos in both study populations were separately analyzed for interobserver reliability by independent coders who were blind to behavioral profiles of individuals. In UVI Austria, the interobserver reliability was excellent both for frequencies (ICC (3, 1) = 0.902, 95% confidence interval [CI] lower, upper = 0.875, 0.923, *F* = 10.208, *p* < .001) and durations and latencies (ICC (3, 1) = 0.940, 95% CI lower, upper = 0.928, 0.951, *F* = 16.773, *p* < .001). In BBFS Brazil, the interobserver reliability was moderate for frequencies (ICC (3, 1) = 0.541, 95% CI lower, upper = 0.430, 0.630, *F* = 2.176, *p* < .001) and excellent for durations and latencies (ICC (3, 1) = 0.972, 95% CI lower, upper = 0.965, 0.978, *F* = 35.998, *p* < .001), following Koo and Li (2016).

We followed Massen et al. (2013) and our previous report (Šlipogor et al., 2016), first testing for short‐term temporal repeatability of behavioral variables, between the first and the second test session, and separately for PTB1 (Šlipogor et al., 2016), PTB2 (Table S2) and the wild sample (Table S3), by using intra‐class correlation coefficients (ICC (3, 1)). We calculated individual mean values over those two repetitions per testing battery (i.e., separately for PTB1, PTB2, and wild sample) for significantly repeatable variables (*p* < .05), and those with a Cronbach's *α* > .5 in wild sample. Then, we tested for contextual consistency of these variables using ICCs (Tables S4–S5). We entered all contextually consistent variables, those with an Cronbach's *α* > .5 (i.e., that showed a significant trend), and the rest of variables that were temporally consistent, but not contextually consistent into a principal component analysis (PCA) (Tables 3 and 4), to get the most comprehensive selection of variables from tests. The PCA‐solution was Varimax‐rotated and variable loadings >0.4 and <−0.4 were considered salient (cf. Konečná et al., 2012). Varimax rotation is an orthogonal type of rotation that rotates the original factors to maximize the sum of the variance of the squared loadings by minimizing complexity of factors (i.e., by making high loadings of variables higher and low loadings lower for every factor) (Tabachnik & Fidell, 2013). A direct Oblimin rotation, an oblique type of rotation that simplifies factors by minimizing cross‐products of loadings (Tabachnick & Fidell, 2013) corroborated the independence of the components. We combined several different approaches to assess the number of components to retain in the component solution and to inspect its robustness (Morton & Altschul, 2019: eigenvalues (>1), scree plots and Horn's Parallel Analysis with 1000 iterations). We calculated component scores from the PCA components with a regression method (Massen et al., 2013). This method is a least squares regression approach to estimate factor scores, that uses factor score coefficients, rather than component loadings as weights in equation (Thurstone, 1935). In particular, the component loadings are adjusted to consider the initial correlations between variables, and when doing so, differences in measurement units and variable variances are stabilized (Field, 2018). The regression method produces standardized component scores (i.e., with a mean equal to 0 and a variance equal to the squared multiple correlation between the estimated component scores and the true component values), and essentially predicts the “location” of each individual on the component, that is, the component score represents a composite score for each individual on a particular component (DiStefano et al., 2009; Field, 2018; Tabachnick & Fidell, 2013). Due to our relatively small sample size, we repeated all analyses with a regularized exploratory factor analysis (REFA), following procedure described in Úbeda et al. (2019) and Úbeda and Llorente (2015). Namely, we used unweighted least squares and Quartimax rotation for factor extraction. REFA is a technique recently developed to assess factor structure when a sample size is relatively small (<50 cases) (Jung, 2013; Jung & Lee, 2011) and has been implemented successfully in primate personality research (e.g., Garai et al., 2016; Konečná et al., 2012; Masilkova et al., 2018; Wilson et al., 2018). We compared the REFA‐derived structures to PCA‐derived structures by inspection of variable loadings and by correlating personality components from PCA to their corresponding components in REFA (e.g., Exploration–Avoidance in PCA with Exploration–Avoidance in REFA). As structures and variable loadings highly corresponded to each other and components were highly correlated (see Tables S6–S7), we proceeded with analyses using the PCA‐derived structures. In total, we ran separate PCAs for PTB1, PTB2, and the wild sample. We used Generalized Linear Mixed Models (GLMMs) to assess the effect of family group and age (continuous age: ACF Vienna [PTB1 and PTB2]; and age class: BBFS Brazil) on the derived component scores. In the initial full models, we included group, age, and their two‐way interaction as fixed factors, and then to find the best models, we used a backward stepwise approach based on the model comparisons with corrected Akaike Information Criterion (cAIC). We used Kruskal–Wallis, Mann–Whitney U‐tests and Spearman's correlations as post‐hoc tests. In both the PTB2 and the wild sample, we additionally checked whether personality traits differed depending on the social (i.e., breeding) status of subjects.

**Table 3:** Personality structure of common marmosets in UVI Austria

	Component				
	Exploration–Avoidance	Boldness–Shyness	Stress/Activity	Fourth	Communalities
Eigenvalues	5.524*	2.978*	2.127*	1.496	
Percentiles	2.946	2.391	2.023	1.752	
% Variance	36.83	19.86	14.18	9.98	
Ground^D^ (GA, NO, FUR)	**0.934**				0.913
Manipulation Target^D^ (FUR)	**0.827**				0.778
Proximity^D^ (GA, NO, NF, FUR)	**0.754**	−0.492			0.840
Food Calls^F^ (FUR)	**0.710**				0.544
Body^L^ (GA, NO, NF, FUR, P)	**−0.700**	0.578			0.853
Touch^L^ (GA, NF, FUR)	**−0.894**				0.895
Enter^L^ (GA, NO, NF, FUR, P)		**0.847**			0.842
Distance^D^ (GA, NO, NF, FUR, P)		**0.815**			0.806
Focus^D^ (GA, NO, NF, FUR, P)		**−0.927**			0.906
Locomotion^D^ (GA, NO, NF, FUR, P)			**0.871**		0.906
Compartment Alternations^F^ (GA, NO, NF, FUR, P)			**0.854**		0.821
Stress Behavior^F^ (NO, NF, P)			**0.761**		0.598
Vigilance Calls^F^ (GA, NO, P)			**0.716**		0.733
Manipulation Target^D^ (NF)				**0.926**	0.864
Contact Calls^F^ (NO, FUR, P)				**0.900**	0.826

*Note*: Variable loadings in PCA, together with parallel analysis results. Varimax rotation with Kaiser normalization. Loadings >0.4 and < −0.4 were considered as salient, and high loadings >0.7 and <−0.7 are indicated in boldface. Communalities indicate a proportion of each variable's variance that can be explained by the principal components. Eigenvalues indicate eigenvalues as obtained by the PCA. Percentiles indicate eigenvalues as obtained by parallel analysis with 1000 iterations. *Eigenvalues larger than percentiles.

Abbreviations: FUR, Foraging Under Risk; GA, General Activity; NO, Novel Object; NF, Novel Food; P, Predator; PCA, principal component analysis.

**Table 4:** Personality structure of common marmosets in BBFS Brazil

	Component					
	Exploration–Avoidance	Boldness–Shyness in Foraging	Boldness–Shyness in Predation	Sociability–Aggressiveness	Stress/Vigilance	Communalities
Eigenvalues	3.616*	2.712*	1.766	1.507	1.382	
Percentiles	3.260	2.528	2.071	1.704	1.418	
% Variance	27.81	20.86	13.58	11.59	10.63	
Focus Target^D^ (NF, SR)	**0.903**					0.878
Proximity^D^ (NF)	**0.903**					0.928
Distance^D^ (NO, NF)	**−0.894**					0.883
Platform^D^ (NO, NF, P)	**0.813**					0.950
Platform^L^ (GA, NO, SR)		**0.967**				0.972
Return^L^ (SR)		**0.934**				0.972
SUM Calls^F^ (NF)		**0.824**				0.815
Touch^L^ (NO)			**0.941**			0.900
Body^L^ (P)			**0.875**			0.831
Socionegative Initiate^F^ (NO, NF)				**−0.810**		0.723
Sociopositive Initiate^F^ (NO)				**0.767**		0.711
Stress Behavior^F^ (GA)					**0.771**	0.761
SUM Calls^F^ (NO)					**0.763**	0.658

*Note*: Variable loadings in a PCA. Varimax rotation with Kaiser normalization. Loadings >0.4 and <−0.4 were considered as salient, and high loadings >0.7 and <−0.7 are indicated in boldface. Communalities indicate a proportion of each variable's variance that can be explained by the principal components. Eigenvalues indicate eigenvalues as obtained by the PCA, whereas percentiles indicate eigenvalues as obtained by parallel analysis with 1000 iterations. *Eigenvalues larger than percentiles.

Abbreviations: FUR, Foraging Under Risk; GA, General Activity; NO, Novel Object; NF, Novel Food; P, Predator; PCA, principal component analysis.

To answer whether personality structure is consistent across 4 years, we compared the PTB1 and PTB2 for the long‐term temporal consistency of personality traits. We first inspected personality components and their behavioral loadings from PTB1 and PTB2. We then inverted PTB2 component scores that were in opposite directions to corresponding PTB1 component scores (namely, Exploration–Avoidance and Boldness–Shyness), and then, to calculate delta scores (i.e., the scores obtained when subtracting the PTB1 scores from the PTB2 scores) between two test batteries for further analyses, we added a number 4 to all components from both batteries. Then, we used intra‐class correlation analyses (ICC (3, 1)) on subjects tested at both time periods (*N* = 13) to compare both equally *labelled* components (Exploration‐Avoidance in PTB1 and PTB2), and equally *important* components (i.e., those explaining the same amount of variance in data, e.g., the first component in PTB1 and PTB2) and inspected the obtained plots. Finally, we checked whether individuals that changed their breeding status over these 4 years also showed a change in levels of their personality traits, using Mann–Whitney *U*‐tests.

To answer whether personality structures from captivity reflect the structures obtained under natural setting, we inspected loadings of different behaviors on the obtained personality components and differences in personality structure, and discuss it below.

## 3 RESULTS

### 3.1 Short‐term temporal consistency and personality structure in captive population

Short‐term temporal and contextual consistency obtained from PTB2 (see SEM, Tables S2 and S4) was overall higher than in PTB1 (Šlipogor et al., 2016) with the ICC values of temporally repeatable variables ranging from 0.32 (*Body* ^L^ in NO) to 0.96 (*Contact Calls* ^F^ in NO). After averaging the values from the two repetitions of variables that were temporally and/or contextually consistent (described above), inspecting the eigenvalues and the scree plot, we extracted four personality components from PTB2, which together explained 80.84% of the variance. The first component (36.83%) consisted of exploratory behavior and manipulation of stimuli, so we labeled it “Exploration–Avoidance”. The second component (19.86%) consisted of variables related to bold tendencies and staying in close physical and visual contact to the stimulus, so we labeled it “Boldness–Shyness”. The third component (14.18%) mostly consisted of behaviors related to stress and activity, thus we labelled it “Stress/Activity”. The fourth component (9.98%) consisted of only two variables, however, as percentiles obtained by the parallel analysis for this component were larger than eigenvalues obtained by the PCA, and the personality structure was solid even without it, we discarded it from further analyses (Table 3). REFA structure corroborated PCA structure, whereas the second and third components were reversed (Table S6). The best fitting models on personality component scores of Exploration–Avoidance and Stress/Activity revealed no age effects. In terms of Exploration–Avoidance, the best model was explained by group and an interaction of group and age (*F* = 1.219, df 1,2 = 9,17, *p* = .346), but neither effect was significant: group (*F* = 0.753, df 1,2 = 4,17, *p* = .570), interaction of group and age (*F* = 2.079, df 1,2 = 5,17, *p* = .118). There was a significant interaction effect of group and age (*F* = 5.422, df 1,2 = 4,17, *p* = .005) and a main effect of group on Boldness–Shyness (*F* = 8.097, df 1,2 = 9,17, *p* < .000). In particular, some groups were shyer overall (Pooh, *ß*‐coefficient: −2.486, Ginevra, *ß*‐coefficient: −0.502). These groups also consisted of older individuals in our study population, and these were overall shyer than younger individuals (*r*_s_ = 0.498, *p* = .008). Lastly, there was a significant effect of group on Stress/Activity (*F* = 7.170, df 1,2 = 4,22, *p* < .001). Post‐hoc analyses identified that one group (i.e., Ginevra) had overall significantly lower Stress/Activity factors scores than members of other groups, and in particular of that of group Sparrow (Kruskal–Wallis test; *H* = 16.071, df = 4, *p* = .003; Mann–Whitney *U*‐tests, Holm–Bonferroni corrected *p*‐values [Holm, 1979]: group Pooh vs. group Ginevra *U* = 0.000, *Z* = −2.449, *p* = .056; group Sparrow vs. group Ginevra *U* = 0.000, *Z* = −2.928, *p* = .012; group Veli vs. group Ginevra *U* = 2.000, *Z* = −2.378, *p* = .068; group Kiri vs. group Ginevra *U* = 9.000, *Z* = −2.199, *p* = .112) (Table S8). Breeders and non‐breeders did not differ in terms of their personality traits (Table S10).

### 3.2 Long‐term temporal consistency of personality structure in captive population

**Figure 1:**
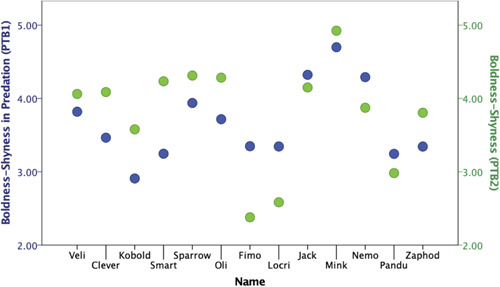
Change in Boldness‐Shyness factor score from PTB1 to PTB2. First six individuals changed their breeding status across 4 years (Veli, Clever, Kobold, Smart, Sparrow, and Oli), whereas eight individuals stayed in the same breeding status (Fimo, Locri, Jack, Mink, Nemo, Pandu, and Zaphod). Blue values depict Boldness‐Shyness factor scores from the PTB1, Green values depict Boldness‐Shynessfactor scores from the PTB2

Overall, personality structure and variable loadings obtained in both test batteries revealed a high similarity in personality traits Boldness–Shyness, Exploration–Avoidance, and Stress/Activity. In PTB1 we found two components indicating bold tendencies, whereas in PTB2, all boldness measurements converged to one component. This Boldness–Shyness (PTB2) corresponded mostly to Boldness–Shyness in Predation (PTB1), in terms of its component loadings. Exploration–Avoidance (PTB2) had loadings of behaviors related to exploration, but was less similar to the previously found Exploration–Avoidance (PTB1). Instead, it highly corresponded to the previously found Boldness–Shyness in Foraging (PTB1). Stress/Activity (PTB2) corresponded to the previously found Stress–Activity (PTB1) component in terms of its variable loadings. We inverted component scores of Exploration–Avoidance and Boldness–Shyness (PTB2) that were in opposite directions to corresponding PTB1 component scores and further transposed all components to consist of positive values only (i.e., by adding a number 4 to all). We found that individuals showed remarkable temporal repeatability across 4 testing years in Exploration–Avoidance (PTB2) and Boldness–Shyness in Foraging (PTB1) (ICC = 0.860, 95% CI lower, upper = 0.542, 0.957, *F* = 7.160, *p* < .001), and in Boldness–Shyness (PTB2) and Boldness–Shyness in Predation (PTB1) (ICC = 0.724, 95% CI lower, upper = 0.096, 0.916, *F* = 3.624, *p* = .017). However, Exploration–Avoidance and Stress/Activity were not consistent with their correspondingly *labelled* components in PTB1 (Exploration–Avoidance: ICC = 0.208, 95% CI lower, upper = −1.594, 0.758, *F* = 1.263, *p* = .346; Stress/Activity: ICC = −1.319, 95% CI lower, upper = −6.599, 0.293, *F* = 0.431, *p* = .920). Finally, we found that the individuals that changed their social status in these 4 years increased their Boldness levels in comparison to those that remained in the same breeding status (Mann–Whitey *U*‐test: *U* = 2.000, *Z* = −2.714, *p* = .007) (Figure 1), and this effect was particularly evident for individuals that changed their status from helpers to breeders (Mann–Whitney *U*‐test: *U* = 2.000, *Z* = −2.517, *p* = .012).

### 3.3 Short‐term temporal consistency and personality structure in wild population

In the wild population we did find short‐term temporal and contextual consistency of behaviors, albeit to a lesser degree than in the captive population (see SEM, Tables S3 and S5), with ICC values of temporally repeatable variables ranging from 0.39 (*Contact Calls* ^F^ in GA) to 0.99 (*Return* ^L^ in SR). After averaging the temporally and/or contextually consistent variables from two testing sessions, we extracted five main personality components, based on their eigenvalues and scree plot test, together explaining 84.48% of the variance. The first component (27.81%) had high loadings of exploratory behavior and focus on the stimuli, thus we labeled it “Exploration–Avoidance”. The second (20.86%) and third components (13.58%) had high loadings of behaviors related to bold tendencies, namely approaches to the platform and the stimulus under foraging conditions (“Boldness–Shyness in Foraging”), and approaches to the predator and novel stimulus (“Boldness–Shyness in Predation”), respectively. The fourth component (11.59%) had high loadings of sociopositive and socionegative behaviors (i.e., in opposite directions), so we labelled it “Sociability–Aggressiveness”. The final component (10.63%) consisted of stress‐related behaviors and calls given in the context of novelty, thus we named it “Stress/Vigilance” (Table 4). PCA structure was corroborated by a REFA structure, but the fourth and fifth component were reversed (Table S7). Parallel analysis results indicated, in contrast, that only two components should be retained in the component solution (namely, Exploration–Avoidance and Boldness–Shyness in Foraging), thereby suggesting that the last three components fall short of contributing to the personality structure in the wild population. However, as all five components had eigenvalues > 1 in both PCA and REFA, and the scree plot test indicated retaining five components in the component solution, we decided to retain all components and to further analyze the five‐component personality structure. However, due to taking a different data reduction approach between the captive and wild data sets, inferences about their structural similarity are limited and thus we need to treat these results with caution.

The best fitting models that aimed to explain individual variation in Exploration–Avoidance component scores revealed a significant group effect (*F* = 10.226, df 1,2 = 4,8, *p* = .003) and a nonsignificant trend of interaction of group and age (*F* = 3.099, df 1,2 = 5,8, *p* = .075). Post‐hoc analyses identified that one group (i.e., Casa) had overall higher Exploration–Avoidance factor scores than members of other groups, but the effect was lost when controlling for multiple testing (Mann–Whitney *U*‐test; Holm–Bonferroni corrected *p*‐values (Holm, 1979); group Azul vs. group Casa *U* = 0.000, *Z* = −2.121, *p* = .136; group Coqueiro vs. group Casa *U* = 0.000, *Z* = −2.309, *p* = .084; group Star Wars vs. group Casa *U* = 0.000, *Z* = −1.061, *p* = 1.000; group Vacas vs. group Casa *U* = 0.000, *Z* = −2.309, *p* = .084). Models conducted on other personality traits did not show any significant effects (Table S9). Furthermore, we found that breeders and helpers significantly differed in terms of their Sociability–Aggressiveness personality traits (Mann–Whitney *U*‐test; Sociability–Aggressiveness: *U* = 11.000, *Z* = −2.577, *p* = .010), namely, breeders showed more socionegative behaviors, and less sociopositive behaviors than other members of the group, but we did not find a difference in any other personality trait in regard to the breeding status (Table S10).

## 4 DISCUSSION

In this study, we investigated long‐term temporal consistency of captive common marmoset personality structure across 4 years, and whether the captive personality structure reflects structure under natural conditions. Temporal consistency of behavioral variables in a repeated personality test battery in captivity and in the personality battery in the wild fell within the higher range of repeatability in animal and primate personality studies in particular (Bell et al., 2009; Freeman & Gosling, 2010). The repeated personality assessment of the captive colony corroborated the previously found personality structure, including Exploration–Avoidance, Boldness–Shyness, and Stress/Activity. Personality structures only slightly differed between the 4 years. In the second personality test battery, we found a smaller number of components than in the first personality test battery, yet their variable loadings were higher than in the previous report, possibly due to the higher number of tested individuals and higher overall temporal and contextual consistency of behavior. These findings support our hypothesis that the personality structure of captive marmosets is relatively stable across years, as long as social conditions remain relatively constant, which might suggest the same underlying mechanism determining the personality structure (Araya‐Ajoy & Dingemanse, 2014). Although monkeys were tested individually, we found a group effect together with an interaction effect of group and age on Boldness–Shyness and Exploration–Avoidance, which mirrors results from previous studies (Koski & Burkart, 2015; Šlipogor et al., 2016); this time, we also found a significant group effect on personality component Stress/Activity, indicating that group‐level similarity may be expressed in several personality axes. Somewhat similar results were found in a study on captive rhesus monkeys, in which inter‐group variation in social behavior was related to group differences in mean level and variation in personality dimension Sociability among group members (Capitanio, 2004).

Exploration–Avoidance and Boldness–Shyness showed a remarkable temporal consistency across 4 years, which supported similar findings of long‐term consistency in personality in other primates (Dutton, 2008; Stevenson‐Hinde et al., 1980a; Weiss et al., 2017), as well as that of the marmosets' consistency in solving extractive foraging tasks across a similar time period (Gunhold et al., 2015). However, we found that a change in breeding status (i.e., from helper to breeder) inside of the family group was linked to the overall increase in the individuals' personality trait Boldness–Shyness (Figure 1). The notion that certain personality traits are connected with particular life‐history outcomes, for example, that higher levels of boldness, exploration, or proactivity in individuals predict the higher status in hierarchy and rise to the breeding status and/or leading status in the group has recently started to be explored in animals (Aplin et al., 2014, Beauchamp, 2000). For example, bold homing pigeons occupy higher ranks in leadership hierarchy and are more likely to have more influence on the direction of collective movement than shy individuals (Sasaki et al., 2018), and field crickets that change their status from subordinate to dominant become bolder, more explorative and more active (Rudin et al., 2017). To our knowledge, this is the first such finding in marmosets, although its causes and consequences remain to be further explored.

The wild population showed a five‐component personality structure, namely Exploration–Avoidance, Boldness–Shyness in Foraging, Boldness–Shyness in Predation, Sociability–Aggressiveness and Stress/Vigilance, which to an extent corroborated the personality structure as obtained with individual behavioral testing in captive marmosets. Intriguingly, it seems that reactions of wild monkeys to novel objects were more reflective of Boldness, than of Exploration, as approaches to a novel object and to a predator loaded on the same component, whereas in captivity this was not the case. This urges caution in drawing strong conclusions on differences between Exploration and Boldness in free‐ranging individuals, at least in the current test paradigm (also see Carter et al., 2012a). We found a significant effect of group and an interaction of group and age on Exploration–Avoidance, giving the first support of the group‐level similarity in Exploration in wild marmosets. However, after controlling for multiple testing, the effect was gone, so we should treat this result with caution. Similarity in personality on the level of the group may promote coordination or cooperation among group members (Planas‐Sitjà et al., 2015), which is particularly beneficial for highly social species with prosocial tendencies and prolonged infant care, maybe as a product of social learning and behavioral convergence (Koski & Burkart, 2015).

The obtained five‐component personality structure, selected based on the eigenvalues > 1 and a scree plot test, may indicate that the wild monkeys have an enlarged personality structure compared to the monkeys in captivity. However, after applying parallel analysis to both PCA‐ and REFA‐derived personality structures, only two components that are most commonly found with behavioral testing, namely, Exploration–Avoidance and Boldness–Shyness, are retained in the factor solution. It is intriguing that different selection methods resulted in such striking variation in personality structure. Perhaps this was due to our relatively small sample size of animals, a low number of cross‐contextually consistent variables that may have rendered an unreliable factor solution, rare socio‐positive and ‐negative behaviors, and behaviors indicative of stress and activity, that did not manage to retain sufficiently strong components after additional analyses.

Both obtained personality structures, the expanded five‐factor and suppressed two‐factor structure, are somewhat different to the captive personality structures, likely because monkeys in the wild were tested together with their family group. The within‐group dynamics of social setting could have either further enhanced or reduced the expression of personality traits. The five‐factor structure contained Sociability–Aggressiveness component, consisting of social behavior and Stress/Vigilance component, consisting of behaviors related to stress and different calls. Socio‐positive and ‐negative behaviors were only seen during group testing, and thus this factor could not have emerged during individual testing in captivity. Interestingly, we found a significant difference in Sociability–Aggressiveness component between breeders and helpers in the wild; in particular, breeders showed more socionegative behaviors, and less sociopositive behaviors than helpers, probably due to higher competition from the breeders for limited food resources during the test situations (De la Fuente et al., 2019). Further, activity and stress are not necessarily always reflecting personality; they can also be a result of behavioral contagion or related mechanisms, allowing behavioral matching with the majority of the group or just with specific group members (e.g., contagious scent marking, Massen et al., 2016), so Stress/Vigilance component might have also been a result of the social testing.

Interestingly, the two‐component structure was remarkably similar to the two‐component personality structure consisting of Boldness and Exploration, from a previous study testing captive common marmosets in a group setting (Koski & Burkart, 2015). Perhaps the highly social character of common marmosets and dependence on their social companions on the one hand leads to their similarity within the group (i.e., even when separated), while on the other hand constrains their full expression of personality traits when tested socially. To assess to which extent the social setting influences personality structure in this species, the future studies should aim to assess personality of the same populations in both social and individual settings. Obtaining the full spectrum of personality structure in this species might further need complementing behavioral testing with other personality assessment methods. For example, one could conduct long‐term behavioral observations or focal follows of individual animals, and/or design special tests (e.g., food‐sharing tests) to gather the full range of social dynamics in the group when foraging and/or sharing limited resources, to reliably capture Sociability or Aggressiveness component.

The study of nonhuman primate personality and its links with fitness, antipredator or foraging behavior has gained momentum (e.g., Blaszczyk, 2017; Carter et al., 2012a; Dammhahn & Almeling, 2012; Perry et al., 2017, and could perhaps become a critical topic in understanding biogeography or speciation, or improving conservation efforts for endangered species (cf. Blaszczyk, 2019; Canestrelli et al., 2016; Ingley & Johnson, 2014; McDougall et al., 2006). In this study, we mainly considered the factor loadings and differences in the found personality components. Ideally, we would have compared the personality structures of captivity and the wild using Procrustes rotation (McCrae et al., 1996) of population‐specific personality structures toward previously established one (e.g., as was done when comparing personality structures in two different species of squirrel monkeys, Wilson et al., 2018). However, given sample differences and procedural differences in test set‐ups, our data did not allow for such analyses. As a consequence our data remain relatively descriptive, but serve the purpose of our study which was to assess long‐term temporal consistency of personality structure in captivity and its ecological validity under natural conditions.

In sum, by using behavioral testing in common marmosets, we showed long‐term consistency of personality structure in captivity and its correspondence to the personality structure under natural conditions; thereby confirming its reliability and, to some extent, ecological validity. Furthermore, we discovered that across years, individuals that changed their social status in the group, increased their bold tendencies.

## CONFLICT OF INTERESTS

The authors declare that there are no conflict of interests.

## Supporting information

Supporting information.

## Data Availability

The data sets generated and/or analyzed during this study are available from the corresponding author on request.
