# Supplementary material for: Temporal consistency and ecological validity of personality structure in common marmosets (Callithrix jacchus): A unifying field and laboratory approach
Source: Am J Primatol. 2021 Jan 19;83(2):e23229. doi: 10.1002/ajp.23229 (PMC7900989; doi:10.1002/ajp.23229)
Supplement: Supplementary file 1 — Supporting information. [file AJP-83-e23229-s001.pdf]

Supplemental Electronic Material

Temporal consistency and ecological validity of personality structure in common marmosets (*Callithrix jacchus*): A unifying field and laboratory approach

Šlipogor, V.*, Massen, J.J.M., Schiel, N., Souto, A. & Bugnyar, T.

*corresponding author

INDEX

Results 2

Captive Population 2

Wild Population 2

References 3

Figure S1. Test Set-up in Captivity 4

Figure S2. Test Set-up in Wild 5

Table S1. Individual Data on Marmosets 6

Table S2. Temporal Repeatability in Captivity 7

Table S3. Temporal Repeatability in Wild 9

Table S4. Contextual Consistency in Captivity 11

Table S5. Contextual Consistency in Wild 12

Table S6. REFA Personality Structure in Captivity 13

Table S7. REFA Personality Structure in Wild 14

Table S8. Best-fitting Models (GLMMs) of Personality Traits in Captivity 15

Table S9. Best-fitting Models (GLMMs) of Personality Traits in Wild 16

Table S10. Personality Traits and Breeding Status in Captivity and Wild 18

Table S11. PCA Personality Structure in Captivity, in PTB1 19

Results

Captive population (UVI Austria)

We found that 58 out of 83 behavioral variables measured in all tests were significantly temporally repeatable across the two test sessions in PTB2 (Table S2), which was higher than in PTB1, in our previous report (Šlipogor et al. 2016). We averaged these temporally repeatable variables and assessed their contextual consistency across different tests (Table S4). We found that some of the variables showed significant cross-experimental consistency (e.g., *Enter*^{L} across GA, NO, NF, FUR and P, ICC=0.651, 95% CI lower, upper=0.495, 0.794, F=10.330, P<0.001) and for these variables we again calculated a single mean value across tests, whereas other ones (e.g., *Manipulation*^{D} was not consistent over NF and FUR) were kept for further analyses as unaveraged scores. To investigate how these variables were associated with each other as personality components, we entered them into a principal component analysis (PCA). At first, we entered 20 variables (all of which were temporally consistent, while 13 of them were also contextually consistent). Five of them (*Self-Grooming*^{F}, *Inspection Cage*^{F}, *Ingestion-Related Behaviour*^{F}, *Inspection Lychee*^{F}, *Route*) were then left out from the analyses in a stepwise manner, because of their lower loadings and unreliable factor solution in initial trial runs. In the final PCA, we entered 15 temporally repeatable variables (out of which 13 were contextually consistent). The analyses indicated appropriate sampling adequacy (Kaiser–Meyer–Olkin measure, KMO=0.618; Bartlett's Test of Sphericity, P<0.001), and all variables had communality estimates >0.598 (*Stress Behavior*^{F}). We extracted four personality components, based on eigenvalues (>1) and scree plot tests, which together explained 80.84% of the variance. Parallel analysis, however, indicated retaining first three components in the component solution (Table 3).

Wild population (BBFS Brazil)

We found that 27 out of 92 behavioral variables measured in all tests were significantly repeatable across the two test sessions (Table S3). We calculated mean values of temporally repeatable

variables and those that showed a trend (with a Cronbach's α>0.5) and tested their contextual consistency in different tests (Table S5). Some of the variables showed significant cross-experimental consistency (e.g., *Platform*^{L} across GA, NO and SR, ICC=0.897, 95% CI lower, upper=0.791, 0.956, F=27.054, P<0.001), some showed a trend, with a Cronbach's α>0.5, and these variables were averaged across tests. The other non-contextually consistent variables were kept as unaveraged scores. First, we entered 17 variables (all temporally consistent, while 6 of them were also contextually consistent) into a PCA to investigate how they are associated with each other as personality components. Four of them (*Stress SUM*^{F} in NO, *Body*^{L} in NF, *Locomotion*^{D} in SR, *Compartment Alternations*^{F} in SR and P) were left out from the analyses in a stepwise manner, because of their lower loadings and unreliable factor solution in initial trial runs. In the final PCA, we entered 13 temporally repeatable variables (4 contextually consistent). The analyses indicated appropriate sampling adequacy (Kaiser–Meyer–Olkin measure, KMO=0.494; Bartlett's Test of Sphericity, P<0.001), and all variables had communality estimates >0.658 (*SUM Calls*^{F} in NO). We extracted five main personality components, based on eigenvalues (>1) and scree plot tests, together explaining 84.48% of the variance. However, parallel analysis results indicated that only two components should be considered for the full personality structure (namely, Exploration-Avoidance and Boldness-Shyness in Foraging). We nevertheless decided to retain and further inspect all components, but we thus need to treat these results with caution (Table 4).

References

Šlipogor, V., Gunhold-de Oliveira, T., Tadić, Z., Massen, J. J. M., & Bugnyar, T. (2016). Consistent inter-individual differences in common marmosets (*Callithrix jacchus*) in Boldness-Shyness, Stress-Activity, and Exploration-Avoidance. *American Journal of Primatology, 78*(9), 961-973.https://doi.org/10.1002/ajp.22566

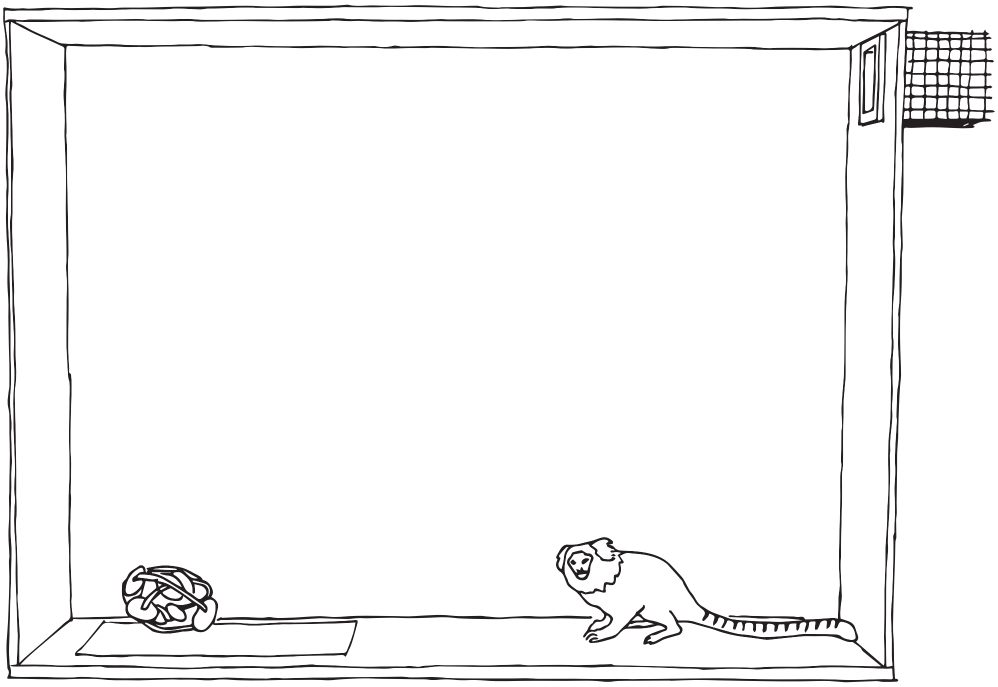

Figure S1 Frontal view of the experimental cage with the NO set-up in UVI Austria.

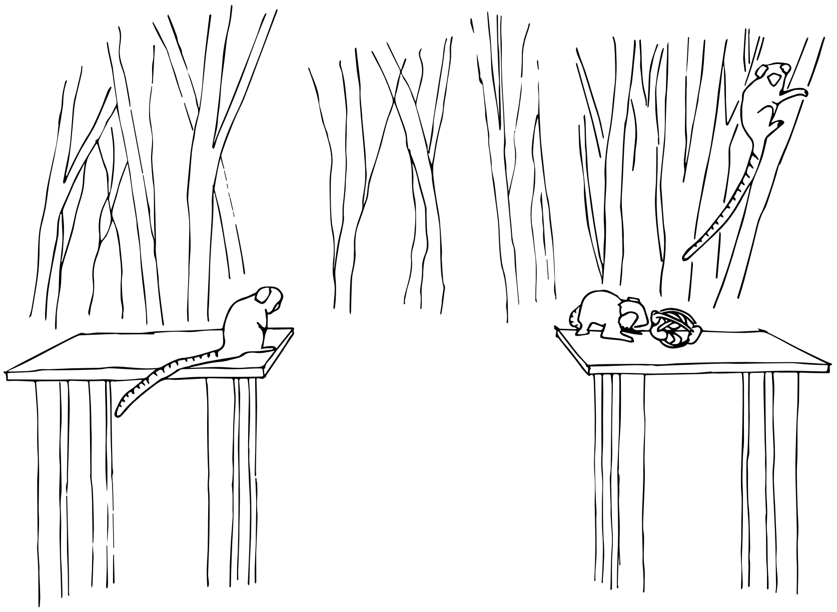

Figure S2 Frontal view of the two wooden platforms with the NO set-up in BBFS Brazil.

Table S1. Individual Data on Marmosets Tested in this Study. Test location, group membership, name, age (UVI Austria; in years), approximate age (BBFS Brazil; A=adult, SA=subadult, J=juvenile), sex (M=male, F=female), social status (B=breeder, H=helper).

Test Location	Group	Individual	Age	Sex	Status
UVI Austria	Ginevra	Ernesto	11	M	B
		Ginevra	12	F	B
		Matilda	1	F	H
		Melvin	1	M	H
		Vincent	3	M	H
		Valentino	2	M	H
		Vento	6	M	H
		Vero	2	M	H
	Kiri	Aurora	3	F	H
		Jack	9	M	H
		Luna	2	F	H
		Mink	10	M	H
		Nemo	10	F	H
		Oli	10	F	B
		Zaphod	13	M	B
	Pooh	Fimo	13	M	H
		Locri	12	M	H
		Pandu	12	F	H
	Sparrow	Kobold	10	M	H
		Nala	0.5	F	H
		Simba	0.5	M	H
		Smart	6	M	B
		Sparrow	9	F	B
	Veli	Blinky Bill	0.5	M	H
		Clever	6	M	B
		Veli	11	F	B
		Wall-E	0.5	M	H
BBFS Brazil	Casa	Metuzalem	A	M	B
		Baltazar	A	M	H
		Amy	A	F	B
		Kevin	J	M	H
	Coqueiro	Sansa	A	F	B
		Dedinho	A	M	B
		Arya	SA	F	H
		Carmen	SA	F	H
	Azul	Azul	A	M	B
		Nena	A	F	B
		Emo	J	M	H
	Star Wars	Leia	A	F	B
		Luke	A	M	B
		Darth Vader	J	M	H
	Vacas	Branca	A	F	B
		Fogo	A	M	B
		Zustje	J	F	H
		Broertje	J	M	H

Table S2. Temporal Repeatability in Captivity (UVI Austria). Summary of all behavioral variables and their temporal consistency as intra-class correlation (ICC (3,1)) with 95% confidence intervals. Frequencies are noted with a letter ‘F’ in superscript (^{F}), durations with a letter ‘D’ in superscript (^{D}) and latencies with a letter ‘L’ in superscript (^{L}). The significantly repeatable behavioral variables are shown in bold. Note that in NF and FUR tests, additional variables were measured; namely *Ingestion-Related Behavior^{F}* in NF, and *Ingestion-Related Behavior^{F}*, *Inspection Lychee^{F}*, and *Route* in FUR.

Test	Behavioral Variable	Cronbach's α	ICC (3,1)	95 % CI lower, upper	F, P
GA	**Enter^{L}**	**0.879**	**0.784**	**0.581, 0.896**	**8.275, <0.000**
	**Body^{L}**	**0.781**	**0.640**	**0.350, 0.818**	**4.562, <0.000**
	**Touch^{L}**	**0.718**	**0.561**	**0.236, 0.773**	**3.552, 0.001**
	**Vigilance Calls^{F}**	**0.520**	**0.351**	**-0.026, 0.641**	**2.084, 0.033**
	Contact Calls^{F}	0.379	0.234	-0.154, 0.559	1.610, 0.116
	Food Calls^{F}	-0.223	-0.100	-0.457, 0.284	0.817, 0.694
	Self-Grooming^{F}	-0.267	-0.118	-0.471, 0.268	0.789, 0.725
	Stress Behavior^{F}	0.236	0.134	-0.253, 0.484	1.309, 0.248
	Inspection Cage^{F}	0.076	0.040	-0.339, 0.407	1.082, 0.421
	**Compartment Alternations^{F}**	**0.769**	**0.625**	**0.328, 0.810**	**4.335, <0.000**
	**Locomotion^{D}**	**0.844**	**0.730**	**0.490, 0.867**	**6.419, <0.000**
	**Proximity^{D}**	**0.747**	**0.596**	**0.286, 0.793**	**3.954, <0.000**
	**Ground^{D}**	**0.666**	**0.499**	**0.154, 0.736**	**2.991, 0.003**
	**Distance^{D}**	**0.879**	**0.784**	**0.580, 0.895**	**8.256, <0.000**
	**Focus^{D}**	**0.794**	**0.659**	**0.378, 0.828**	**4.858, <0.000**
	Manipulation$^{D\#}$	/	/	/	/
NO	**Enter^{L}**	**0.876**	**0.779**	**0.571, 0.893**	**8.044, <0.000**
	**Body^{L}**	**0.485**	**0.320**	**-0.061, 0.620**	**1.940, 0.049**
	Touch^{L}	-0.026	-0.013	-0.385, 0.363	0.975, 0.526
	**Vigilance Calls^{F}**	**0.780**	**0.640**	**0.349, 0.818**	**4.550, <0.000**
	**Contact Calls^{F}**	**0.979**	**0.959**	**0.913, 0.981**	**48.346, <0.000**
	Food Calls^{F}	0.342	0.206	-0.182, 0.539	1.520, 0.146
	Self-Grooming^{F}	-0.241	-0.108	-0.463, 0.278	0.806, 0.707
	**Stress Behavior^{F}**	**0.713**	**0.554**	**0.227, 0.768**	**3.480, 0.001**
	**Inspection Cage^{F}**	**0.503**	**0.336**	**-0.043, 0.631**	**2.012, 0.040**
	**Compartment Alternations^{F}**	**0.951**	**0.906**	**0.806, 0.956**	**20.382, <0.000**
	**Locomotion^{D}**	**0.730**	**0.575**	**0.256, 0.781**	**3.704, 0.001**
	**Proximity^{D}**	**0.693**	**0.530**	**0.195, 0.754**	**3.254, 0.002**
	**Ground^{D}**	**0.604**	**0.433**	**0.070, 0.694**	**2.525, 0.011**
	**Distance^{D}**	**0.674**	**0.508**	**0.166, 0.741**	**3.068, 0.003**
	**Focus^{D}**	**0.601**	**0.429**	**0.066, 0.692**	**2.503, 0.011**
	Manipulation^{D}	0.087	0.045	-0.334, 0.412	1.095, 0.409
NF	**Enter^{L}**	**0.682**	**0.518**	**0.178, 0.747**	**3.147, 0.002**
	**Body^{L}**	**0.764**	**0.618**	**0.318, 0.806**	**4.239, <0.000**
	**Touch^{L}**	**0.653**	**0.485**	**0.136, 0.727**	**2.883, 0.004**
	Vigilance Calls^{F}	0.135	0.073	-0.310, 0.435	1.157, 0.357
	Contact Calls^{F}	0.335	0.201	-0.187, 0.535	1.504, 0.152
	Food Calls^{F}	0.012	0.006	-0.369, 0.379	1.012, 0.488
	Self-Grooming^{F}	0.021	0.011	-0.365, 0.383	1.021, 0.479
	**Stress Behavior^{F}**	**0.715**	**0.556**	**0.230, 0.770**	**3.505, 0.001**
	**Inspection Cage^{F}**	**0.557**	**0.386**	**0.014, 0.664**	**2.259, 0.021**
	**Compartment Alternations^{F}**	**0.652**	**0.484**	**0.134, 0.726**	**2.875, 0.005**
	**Locomotion^{D}**	**0.813**	**0.684**	**0.417, 0.843**	**5.338, 0.000**
	**Proximity^{D}**	**0.518**	**0.350**	**-0.028, 0.640**	**2.075, 0.034**
	Ground^{D}	0.434	0.278	-0.108, 0.590	1.768, 0.076
	**Distance^{D}**	**0.633**	**0.463**	**0.108, 0.714**	**2.728, 0.006**

	**Focus[D]**	**0.615**	**0.444**	**0.084, 0.701**	**2.595, 0.009**
	**Manipulation[D]**	**0.794**	**0.659**	**0.378, 0.829**	**4.865, <0.000**
	Ingestion-Related Behavior[F]	0.145	0.078	-0.304, 0.439	1.170, 0.346
FUR	**Enter[L]**	**0.697**	**0.534**	**0.201, 0.757**	**3.295, 0.002**
	**Body[L]**	**0.938**	**0.884**	**0.762, 0.945**	**16.253, <0.000**
	**Touch[L]**	**0.933**	**0.874**	**0.744, 0.941**	**14.917, <0.000**
	Vigilance Calls[F]	0.427	0.272	-0.114, 0.586	1.747, 0.081
	**Contact Calls[F]**	**0.573**	**0.402**	**0.033, 0.674**	**2.344, 0.017**
	**Food Calls[F]**	**0.934**	**0.877**	**0.749, 0.942**	**15.266, <0.000**
	Self-Grooming[F]	-0.261	-0.115	-0.469, 0.270	0.793, 0.720
	Stress Behavior[F]	0.269	0.155	-0.232, 0.500	1.367, 0.215
	Inspection Cage[F]	0.436	0.279	-0.106, 0.591	1.773, 0.075
	**Compartment Alternations[F]**	**0.572**	**0.400**	**0.031, 0.673**	**2.336, 0.017**
	**Locomotion[D]**	**0.775**	**0.633**	**0.339, 0.814**	**4.443, <0.000**
	**Proximity[D]**	**0.921**	**0.854**	**0.705, 0.931**	**12.666, <0.000**
	**Ground[D]**	**0.947**	**0.900**	**0.793, 0.953**	**18.967, <0.000**
	**Distance[D]**	**0.914**	**0.841**	**0.682, 0.924**	**11.613, <0.000**
	**Focus[D]**	**0.793**	**0.657**	**0.376, 0.828**	**4.837, <0.000**
	**Manipulation[D]**	**0.894**	**0.808**	**0.623, 0.908**	**9.439, <0.000**
	**Ingestion-Related Behavior[F]**	**0.698**	**0.536**	**0.202, 0.758**	**3.308, 0.002**
	**Inspection Lychee[F]**	**0.973**	**0.948**	**0.888, 0.976**	**37.108, <0.000**
	**Route**	**0.802**	**0.669**	**0.393, 0.834**	**5.041, <0.000**
P	**Enter[L]**	**0.916**	**0.845**	**0.688, 0.926**	**11.881, <0.000**
	**Body[L]**	**0.578**	**0.406**	**0.038, 0.677**	**2.367, 0.016**
	Touch[L#]	/	/	/	/
	**Vigilance Calls[F]**	**0.775**	**0.633**	**0.339, 0.814**	**4.443, <0.000**
	**Contact Calls[F]**	**0.820**	**0.695**	**0.434, 0.848**	**5.554, <0.000**
	Food Calls[F]	-0.230	-0.103	-0.459, 0.282	0.813, 0.699
	Self-Grooming[F]	-0.360	-0.152	-0.498, 0.235	0.736, 0.781
	**Stress Behavior[F]**	**0.576**	**0.405**	**0.037, 0.676**	**2.361, 0.016**
	**Inspection Cage[F]**	**0.781**	**0.640**	**0.350, 0.818**	**4.557, <0.000**
	**Compartment Alternations[F]**	**0.882**	**0.789**	**0.588, 0.898**	**8.471, <0.000**
	**Locomotion[D]**	**0.785**	**0.646**	**0.359, 0.822**	**4.656, <0.000**
	Proximity[D]	0.094	0.049	-0.331, 0.416	1.104, 0.402
	Ground[D]	0.060	0.031	-0.347, 0.400	1.064, 0.438
	**Distance[D]**	**0.839**	**0.723**	**0.478, 0.863**	**6.218, <0.000**
	**Focus[D]**	**0.891**	**0.803**	**0.613, 0.905**	**9.143, <0.000**
	Manipulation[D#]	/	/	/	/

Note that for these behavioral variables we were unable to compute the ICC values, due to zero variance.

Table S3. Temporal Repeatability in Wild (BBFS Brazil). Summary of all behavioral variables and their temporal consistency as intra-class correlation (ICC (3,1)) with 95% confidence intervals. Frequencies are noted with a letter 'F', durations with a letter 'D' and latencies with a letter 'L' in superscript. The significantly repeatable behavioral variables are shown in bold, variables that show a trend (i.e., Cronbach's α>0.5) are shown in bold italic. Note that in NF and SR, additional variables were measured; namely *Nb Eaten Target*^{F} in NF and *Return*^{L} in SR.

Test	Behavioral Variable	Cronbach's α	ICC (3,1)	95 % CI lower, upper	F, P
GA	Compartment Alternations^{F}	-0.154	-0.071	-0.510, 0.397	0.867, 0.614
	**Stress Behavior^{F}**	**0.622**	**0.452**	**-0.005, 0.752**	**2.648, 0.026**
	Self-Grooming^{F}	-0.214	-0.097	-0.529, 0.375	0.824, 0.653
	Vigilance Calls^{F}	0.395	0.246	-0.236, 0.631	1.653, 0.155
	**Contact Calls^{F}**	**0.565**	**0.393**	**-0.076, 0.720**	**2.297, 0.048**
	Food Calls^{F}	-0.047	-0.023	-0.474, 0.437	0.955, 0.537
	SUM Calls^{F}	0.273	0.158	-0.320, 0.573	1.376, 0.259
	Socio-Negative Initiate^{F}	-0.667	-0.250	-0.633, 0.232	0.600, 0.849
	Socio-Positive Initiate$^{F\#}$	/	/	/	/
	**Platform^{L}**	**0.988**	**0.976**	**0.938, 0.991**	**83.882, 0.000**
	Body^{L}	-0.031	-0.015	-0.468, 0.443	0.970, 0.525
	Touch$^{L\#}$	/	/	/	/
	Locomotion^{D}	0.399	0.249	-0.233, 0.633	1.663, 0.152
	Platform^{D}	-0.095	-0.046	-0.491, 0.419	0.913, 0.573
	Proximity^{D}	-0.697	-0.259	-0.639, 0.223	0.589, 0.857
	Distance^{D}	0.029	0.015	-0.444, 0.467	1.030, 0.476
	Focus^{D}	0.106	0.056	-0.410, 0.499	1.119, 0.410
	Manipulation$^{D\#}$	/	/	/	/
NO	Compartment Alternations^{F}	0.349	0.212	-0.270, 0.609	1.537, 0.192
	**Stress Behavior^{F}**	**0.605**	**0.433**	**-0.028, 0.742**	**2.528, 0.032**
	Self-Grooming^{F}	0.335	0.201	-0.280, 0.602	1.504, 0.204
	**Vigilance Calls^{F}**	**0.671**	**0.504**	**0.063, 0.781**	**3.035, 0.014**
	**Contact Calls^{F}**	**0.599**	**0.427**	**-0.035, 0.739**	**2.492, 0.034**
	**Food Calls^{F}**	**0.675**	**0.510**	**0.070, 0.783**	**3.079, 0.013**
	**SUM Calls^{F}**	**0.579**	**0.407**	**-0.059, 0.728**	**2.375, 0.042**
	**Socio-Negative Initiate^{F}**	**0.839**	**0.722**	**0.397, 0.886**	**6.200, 0.000**
	Socio-Positive Initiate^{F}	***0.518***	***0.349***	***-0.126, 0.694***	***2.074, 0.071***
	**Platform^{L}**	**0.574**	**0.403**	**-0.065, 0.725**	**2.349, 0.044**
	Body^{L}	0.431	0.275	-0.207, 0.649	1.757, 0.128
	Touch^{L}	***0.523***	***0.354***	***-0.121, 0.697***	***2.097, 0.068***
	Locomotion^{D}	0.443	0.284	-0.197, 0.655	1.794, 0.119
	Platform^{D}	***0.510***	***0.343***	***-0.134, 0.690***	***2.043, 0.075***
	Proximity^{D}	-0.474	-0.192	-0.595, 0.289	0.678, 0.784
	**Distance^{D}**	**0.621**	**0.451**	**-0.006, 0.752**	**2.640, 0.026**
	Focus^{D}	0.260	0.150	-0.328, 0.567	1.352, 0.270
	Manipulation^{D}	0.385	0.238	-0.244, 0.626	1.625, 0.163
NF	Compartment Alternations^{F}	0.342	0.206	-0.275, 0.605	1.519, 0.199
	Nb Eaten Target^{F}	0.157	0.085	-0.385, 0.521	1.187, 0.364
	Stress Behavior^{F}	0.120	0.064	-0.403, 0.505	1.137, 0.397
	Self-Grooming^{F}	-0.353	-0.150	-0.567, 0.328	0.739, 0.730
	**Vigilance Calls^{F}**	**0.788**	**0.651**	**0.277, 0.853**	**4.725, 0.001**
	Contact Calls^{F}	0.365	0.223	-0.259, 0.616	1.574, 0.180
	Food Calls^{F}	0.118	0.062	-0.405, 0.504	1.133, 0.400
	**SUM Calls^{F}**	**0.697**	**0.535**	**0.105, 0.796**	**3.298, 0.009**
	**Socio-Negative Initiate^{F}**	**0.710**	**0.550**	**0.126, 0.804**	**3.445, 0.007**
	Socio-Positive Initiate$^{F\#}$	/	/	/	/
	Platform^{L}	0.488	0.322	-0.156, 0.678	1.951, 0.089

	**Body[L]**	**0.615**	**0.444**	**-0.014, 0.748**	**2.599, 0.028**
	Touch[L]	0.482	0.318	-0.161, 0.675	1.931, 0.093
	Locomotion[D]	-0.375	-0.158	-0.572, 0.321	0.727, 0.741
	**Platform[D]**	**0.894**	**0.808**	**0.557, 0.923**	**9.404, 0.000**
	**Proximity[D]**	**0.836**	**0.718**	**0.390, 0.884**	**6.088, 0.000**
	**Distance[D]**	**0.922**	**0.855**	**0.655, 0.943**	**12.817, 0.000**
	**Focus[D]**	**0.676**	**0.511**	**0.072, 0.784**	**3.087, 0.013**
	Manipulation[D]	0.354	0.215	-0.267, 0.611	1.547, 0.188
	Compartment Alternations[F]	***0.557***	***0.386***	***-0.084, 0.716***	***2.258, 0.051***
	Stress Behavior[F]	0.396	0.247	-0.235, 0.631	1.655, 0.154
	Self-Grooming[F]	0.350	0.212	-0.269, 0.609	1.539, 0.191
	Vigilance Calls[F]	-0.173	-0.080	-0.516, 0.390	0.853, 0.627
	Contact Calls[F]	-0.124	-0.058	-0.500, 0.408	0.890, 0.594
	**Food Calls[F]**	**0.747**	**0.596**	**0.193, 0.827**	**3.951, 0.004**
	SUM Calls[F]	0.478	0.314	-0.165, 0.673	1.915, 0.095
	Socio-Negative Initiate[F]	0.221	0.125	-0.351, 0.549	1.284, 0.306
	Socio-Positive Initiate[F#]	/	/	/	/
SR	**Platform[L]**	**0.879**	**0.784**	**0.511, 0.913**	**8.259, 0.000**
	Body[L]	-0.044	-0.022	-0.473, 0.438	0.957, 0.535
	Touch[L]	-0.061	-0.030	-0.479, 0.432	0.943, 0.548
	**Return[L]**	**0.999**	**0.998**	**0.995, 0.999**	**977.692, 0.000**
	**Locomotion[D]**	**0.747**	**0.596**	**0.192, 0.827**	**3.948, 0.004**
	Platform[D]	0.098	0.052	-0.413, 0.496	1.109, 0.417
	Proximity[D]	0.497	0.330	-0.147, 0.683	1.986, 0.084
	Distance[D]	0.365	0.223	-0.259, 0.616	1.574, 0.179
	**Focus[D]**	**0.598**	**0.426**	**-0.036, 0.738**	**2.486, 0.034**
	Manipulation[D]	0.332	0.199	-0.282, 0.600	1.497, 0.207
	Compartment Alternations[F]	***0.512***	***0.344***	***-0.132, 0.691***	***2.049, 0.075***
	Stress Behavior[F]	-0.180	-0.083	-0.519, 0.387	0.847, 0.632
	Self-Grooming[F]	-0.395	-0.165	-0.577, 0.314	0.717, 0.750
	Vigilance Calls[F]	-0.829	-0.293	-0.660, 0.188	0.547, 0.888
	Contact Calls[F]	0.361	0.220	-0.262, 0.614	1.564, 0.183
	**Food Calls[F]**	**0.821**	**0.696**	**0.352, 0.874**	**5.572, 0.000**
	SUM Calls[F]	-0.625	-0.238	-0.626, 0.244	0.616, 0.837
	Socio-Negative Initiate[F]	-0.178	-0.082	-0.518, 0.388	0.849, 0.630
	Socio-Positive Initiate[F#]	/	/	/	/
P	Platform[L]	0.416	0.263	-0.219, 0.641	1.712, 0.139
	**Body[L]**	**0.573**	**0.402**	**-0.066, 0.725**	**2.343, 0.044**
	Touch[L#]	/	/	/	/
	Locomotion[D]	0.352	0.213	-0.268, 0.610	1.543, 0.190
	**Platform[D]**	**0.615**	**0.445**	**-0.014, 0.748**	**2.601, 0.028**
	Proximity[D]	0.039	0.020	-0.440, 0.471	1.040, 0.468
	Distance[D]	-1.062	-0.347	-0.693, 0.129	0.485, 0.927
	Focus[D]	-1.218	-0.379	-0.711, 0.093	0.451, 0.945
	Manipulation[D#]	/	/	/	/

Note that for these behavioral variables we were unable to compute the ICC values, due to zero variance.

Table S4. Contextual Consistency in Captivity (UVI Austria) of the same behavioral variables across different tests. Frequencies of behavioral variables are indicated with a letter 'F' (^{F}), durations with a letter 'D' (^{D}) and latencies with a letter 'L' in superscript (^{L}). Significantly consistent variables are depicted in bold. Significantly consistent variables were taken into further analyses as averaged scores, whereas those that did not show consistency were taken separately into further analyses.

Behavioral Variable	Tests	Cronbach's α	ICC	95% CI lower, upper	F, P
**Enter^{L}**	**GA, NO, NF, FUR, P**	**0.903**	**0.651**	**0.495, 0.794**	**10.330, <0.000**
**Body^{L}**	**GA, NO, NF, FUR, P**	**0.799**	**0.443**	**0.269, 0.637**	**4.976, <0.000**
**Touch^{L}**	**GA, NF, FUR**	**0.538**	**0.280**	**0.045, 0.533**	**2.165, 0.009**
**Vigilance Calls^{F}**	**GA, NO, P**	**0.566**	**0.303**	**0.067, 0.553**	**2.302, 0.005**
**Contact Calls^{F}**	**NO, FUR, P**	**0.936**	**0.829**	**0.706, 0.911**	**15.545, <0.000**
Food Calls^{F}	FUR	/	/	/	/
Self-Grooming^{F}	/	/	/	/	/
**Stress Behavior^{F}**	**NO, NF, P**	**0.701**	**0.439**	**0.204, 0.661**	**3.349, <0.000**
**Inspection Cage^{F}**	**NO, NF, P**	**0.620**	**0.353**	**0.115, 0.594**	**2.634, 0.001**
**Compartment Alternations^{F}**	**GA, NO, NF, FUR, P**	**0.885**	**0.606**	**0.442, 0.763**	**8.698, <0.000**
**Locomotion^{D}**	**GA, NO, NF, FUR, P**	**0.884**	**0.603**	**0.439, 0.761**	**8.598, <0.000**
**Proximity^{D}**	**GA, NO, NF, FUR**	**0.739**	**0.414**	**0.220, 0.624**	**3.827, <0.000**
**Ground^{D}**	**GA, NO, FUR**	**0.778**	**0.538**	**0.314, 0.732**	**4.497, <0.000**
**Distance^{D}**	**GA, NO, NF, FUR, P**	**0.864**	**0.559**	**0.389, 0.728**	**7.328, <0.000**
**Focus^{D}**	**GA, NO, NF, FUR, P**	**0.729**	**0.349**	**0.181, 0.555**	**3.685, <0.000**
Manipulation^{D}	NF, FUR	-0.067	-0.033	-0.402, 0.345	0.937, 0.565
Ingestion-Related Behavior^{F}*	FUR	/	/	/	/
Inspection Lychee^{F}*	FUR	/	/	/	/
Route*	FUR	/	/	/	/

* Note that these behavioral variables were initially taken into further analyses, even though they showed only temporal repeatability in one test (FUR). However, together with behavioral variable *Inspection Cage^{F}* they were left out from the principal component analyses in a stepwise manner, because of their lower loadings and unreliable factor solution. Note that behavioral variable *Self-Grooming^{F}* was not taken into further analyses.

Table S5. Contextual Consistency in Wild (BBFS Brazil) of the same behavioral variables across different tests. Frequencies of behavioral variables are indicated with a letter 'F', durations with 'D' and latencies with 'L' in superscript. Significantly consistent variables are in bold, behaviors that show a trend (i.e., Cronbach's α>0.5) are depicted in bold italic. Both significantly consistent variables, and those that showed a trend were taken into further analyses as averaged scores, whereas those that did not show consistency were taken separately into further analyses.

Behavioral Variable	Tests	Cronbach's α	ICC	95% CI lower, upper	F, P
Compartment Alternations^{F}	***SR, P***	***0.555***	***0.384***	***-0.087, 0.714***	***2.245, 0.052***
Stress Behavior^{F}	GA, NO	0.443	0.285	-0.196, 0.655	1.796, 0.119
Self-Grooming$^{F\#}$	/	/	/	/	/
Vigilance Calls$^{F\#}$	NO, NF	0.647	0.478	0.028, 0.766	2.829, 0.019
Contact Calls$^{F\#}$	GA, NO	-0.439	-0.180	-0.587, 0.300	0.695, 0.770
**Food Calls$^{F\#}$**	**NO, SR, P**	**0.853**	**0.659**	**0.411, 0.840**	**6.792, 0.000**
SUM Calls^{F}	NO, NF	0.257	0.148	-0.330, 0.565	1.347, 0.273
**Platform^{L}**	**GA, NO, SR**	**0.963**	**0.897**	**0.791, 0.956**	**27.054, 0.000**
Body^{L}	NF, P	0.265	0.152	-0.326, 0.569	1.360, 0.267
Touch^{L}	NO	/	/	/	/
Return^{L}	SR	/	/	/	/
**Socio-Negative Initiate^{F}**	**NO, NF**	**0.765**	**0.620**	**0.229, 0.839**	**4.259, 0.002**
Socio-Positive Initiate^{F}	NO	/	/	/	/
Locomotion^{D}	SR	/	/	/	/
**Platform^{D}**	**NO, NF, P**	**0.594**	**0.328**	**0.039, 0.630**	**2.464, 0.012**
Proximity^{D}	NF	/	/	/	/
Distance^{D}	***NO, NF***	***0.535***	***0.365***	***-0.108, 0.704***	***2.152, 0.062***
**Focus^{D}**	**NF, SR**	**0.838**	**0.721**	**0.395, 0.886**	**6.166, 0.000**
Manipulation^{D}	/	/	/	/	/

#Note that behavioral variable *Self-Grooming^{F}* did not show any temporal consistency, so was not taken into further analyses. The initial PCA runs showed that the solution without *Vigilance Calls^{F}*, *Contact Calls^{F}* and *Food Calls^{F}* showed a better fit, so only their composite variable, *SUM Calls^{F}*, was taken into further analyses.

Table S6. REFA Personality Structure of Common Marmosets in Captivity (ACF Vienna) in PTB2. Variable loadings in regularized exploratory factor analysis (REFA), unweighted least squares extraction method together with parallel analysis results. Quartimax rotation with Kaiser normalization. Loadings >0.4 and <-0.4 were considered as salient, and high loadings >0.7 and <-0.7 are indicated in bold. Communalities indicate a proportion of each variable's variance that can be explained by the factors. Eigenvalues indicate eigenvalues as obtained by the REFA. Percentiles indicate eigenvalues as obtained by parallel analysis with 1000 iterations. Eigenvalues larger than percentiles are indicated with asterisk (*). Spearman’s correlations indicate correspondence of REFA components with corresponding PCA components.

	**Component**				
	**Exploration-Avoidance**	**Stress/Activity**	**Boldness-Shyness**	**Fourth**	**Communalities**
**Eigenvalues**	5.524*	2.978*	2.127*	1.496	
**Percentiles**	2.946	2.391	2.023	1.752	
**% Variance**	36.83	19.86	14.18	9.98	
**P**	<0.001	<0.001	<0.001	<0.001	
**r_s**	0.936	0.896	0.961	0.937	
Ground^{D} (GA, NO, FUR)	**.945**				0.927
Manipulation Target^{D} (FUR)	**.803**				0.885
Proximity^{D} (GA, NO, NF, FUR)	**.801**				0.893
Food Calls^{F} (FUR)	.605				0.730
Body^{L} (GA, NO, NF, FUR, P)	**-.766**		.466		0.914
Touch^{L} (GA, NF, FUR)	**-.922**				0.934
Enter^{L} (GA, NO, NF, FUR, P)			**.766**		0.854
Distance^{D} (GA, NO, NF, FUR, P)	-.471		**.715**		0.863
Focus^{D} (GA, NO, NF, FUR, P)			**-.917**		0.875
Locomotion^{D} (GA, NO, NF, FUR, P)		**.889**			0.940
Compartment Alternations^{F} (GA, NO, NF, FUR, P)		**.828**			0.913
Stress Behavior^{F} (NO, NF, P)		.640			0.655
Vigilance Calls^{F} (GA, NO, P)		.665			0.725
Manipulation Target^{D} (NF)				**.934**	0.748
Contact Calls^{F} (NO, FUR, P)				**.752**	0.693

Table S7. REFA Personality Structure of Common Marmosets in Wild (BBFS Brazil). Variable loadings in regularized exploratory factor analysis (REFA), unweighted least squares extraction method together with parallel analysis results. Quartimax rotation with Kaiser normalization. Loadings >0.4 and <-0.4 were considered as salient, and high loadings >0.7 and <-0.7 are indicated in bold. Communalities indicate a proportion of each variable's variance that can be explained by the principal components. Eigenvalues indicate eigenvalues as obtained by the REFA. Percentiles indicate eigenvalues as obtained by parallel analysis with 1000 iterations. Eigenvalues larger than percentiles are indicated with asterisk (*). Spearman's correlations indicate correspondence of REFA components with corresponding PCA components.

	**Component**					
	**Exploration-Avoidance**	**Boldness-Shyness in Foraging**	**Boldness-Shyness in Predation**	**Stress/Vigilance**	**Sociability-Aggressiveness**	**Communalities**
**Eigenvalues**	3.616*	2.712*	1.766	1.507	1.382	
**Percentiles**	3.260	2.528	2.071	1.704	1.418	
**% Variance**	27.81	20.86	13.58	11.59	10.63	
**P**	<0.001	<0.001	<0.001	<0.001	<0.001	
**r_s**	0.955	0.728	0.936	0.808	0.697	
Focus Target^{D} (NF, SR)	**.879**					0.803
Proximity^{D} (NF)	**.916**					0.913
Distance^{D} (NO, NF)	**-.863**					0.903
Platform^{D} (NO, NF, P)	**.816**					0.923
Platform^{L} (GA, NO, SR)		**.985**				0.994
Return^{L} (SR)		**.951**				0.993
SUM Calls^{F} (NF)		**.759**				0.814
Touch^{L} (NO)			**.951**			0.878
Body^{L} (P)			**.737**			0.612
Socio-Negative Initiate^{F} (NO, NF)					**-.610**	0.513
Socio-Positive Initiate^{F} (NO)					**.587**	0.495
Stress Behavior^{F} (GA)				**.810**		0.626
SUM Calls^{F}(NO)				.404		0.554

Table S8. Best-fitting Models (GLMMs) of Personality Traits in Captivity (UVI Austria) performed on three components obtained from the PCA analysis: Exploration-Avoidance, Boldness-Shyness, and Stress/Activity. Significant effects are indicated in bold. Reference groups are indicated in parenthesis.

component	corrected model	fixed effects		F	(df1, df2)	β-coefficient	± SE	P
Exploration-Avoidance	group*age, group			1.219	(9, 17)			0.346
		group (5)		0.753	(4, 17)			0.570
			1			3.135	14.600	
			2			-0.361	1.117	
			3			0.728	1.011	
			4			-0.608	1.111	
		group*age (5*age)		2.079	(5, 17)			0.118
			1*age			-0.233	1.181	
			2*age			0.207	0.106	
			3*age			-0.070	0.082	
			4*age			0.261	0.110	
			5*age			0.049	0.097	
Boldness-Shyness	**group*age, group**			**8.097**	**(9, 17)**			**0.000**
		**group (5)**		**5.422**	**(4, 17)**			**0.005**
			1			-2.486	8.145	
			2			1.376	0.623	
			3			-0.502	0.564	
			4			1.250	0.620	
		**group*age (5*age)**		**9.695**	**(5, 17)**			**0.000**
			1*age			0.405	0.659	
			2*age			-0.025	0.059	
			3*age			0.305	0.046	
			4*age			-0.017	0.061	
			5*age			0.092	0.054	
Stress/Activity	**group**			**7.170**	**(4, 22)**			**0.001**
		**group (5)**		**7.170**	**(4, 22)**			**0.001**
			1			0.692	0.494	
			2			1.055	0.419	
			3			-0.982	0.371	
			4			-0.067	0.449	

Table S9. Best-fitting Models (GLMMs) of Personality Traits in Wild (BBFS Brazil) performed on five components obtained from the PCA analysis: Exploration-Avoidance, Boldness-Shyness in Foraging, Boldness-Shyness in Predation, Sociability-Aggressiveness and Stress/Vigilance. Significant effects are indicated in bold. Reference groups are indicated in parenthesis.

component	corrected model	fixed effects		F	(df1, df2)	β-coefficient	± SE	P
Exploration-Avoidance	**group, group*age**			**6.164**	**(9, 8)**			**0.009**
		**group (5)**		**10.226**	**(4, 8)**			**0.003**
			1			0.185	0.634	
			2			2.363	0.634	
			3			-0.012	0.518	
			4			2.479	0.634	
		group*age		3.099	(5, 8)			0.075
		(group=1)*(age=3)	1*1			0.507	0.634	
		(group=2)*(age=3)	2*1			-0.484	0.598	
		(group=3)*(age=2)	3*1			-0.222	0.518	
		(group=4)*(age=3)	4*1			-2.318	0.634	
		(group=5)*(age=3)	5*1			0.414	0.518	
Boldness-Shyness in Foraging	group, group*age			1.107	(9, 8)			0.448
		group (5)		1.765	(4, 8)			0.229
			1			-0.145	1.192	
			2			0.370	1.192	
			3			-0.021	0.973	
			4			1.298	1.192	
		group*age		0.145	(5, 8)			0.976
		(group=1)*(age=3)	1*1			0.213	1.192	
		(group=2)*(age=3)	2*1			-0.046	1.123	
		(group=3)*(age=2)	3*1			0.048	0.973	
		(group=4)*(age=3)	4*1			0.973	1.192	
		(group=5)*(age=3)	5*1			0.141	0.973	
Boldness-Shyness in Predation	group*age			2.303	(9, 8)			0.127
		group*age ((group=5)*(age=3))		2.303	(9, 8)			0.127
			1*1			-0.344	0.769	
			1*3			-0.467	0.942	
			2*1			-0.422	0.702	
			2*3			0.040	0.942	
			3*1			-1.400	0.769	
			3*2			-1.063	0.769	
			4*1			-0.351	0.769	
			4*3			-2.938	0.942	
			5*1			-2.128	0.769	
Sociability-Aggressiveness	age, group*age			2.697	(9, 8)			0.089
		age (3)		7.253	(2, 8)			0.016
			1			-1.294	0.726	
			2			0.897	0.726	

		group*age		1.730	(7, 8)			0.229
		(group=5)*(age=1); (group=3)*(age=2)	1*1			0.573	0.726	
			2*1			1.163	0.663	
			3*1			0.957	0.726	
			4*1			1.106	0.726	
		(group=5)*(age=3)	1*3			2.483	0.889	
			2*3			0.437	0.889	
			4*3			0.311	0.889	
	group*age			1.562	(9, 8)			0.271
		group*age ((group=5)*(age=3))		1.562	(9, 8)			0.271
			1*1			-0.218	0.878	
			1*3			1.054	1.075	
			2*1			-0.765	0.801	
Stress/Vigilance			2*3			-1.743	1.075	
			3*1			-1.128	0.878	
			3*2			-0.963	0.878	
			4*1			0.717	0.878	
			4*3			1.011	1.075	
			5*1			-0.162	0.878	

Table S10. Comparisons of Personality Components and Breeding Status in Common Marmosets in UVI Austria and BBFS Brazil, using Mann-Whitney U-tests. Significant differences in personality components between individuals of different breeding status are indicated in bold.

**Study Site**	**Component**	**Breeding Status**		
		**U**	**Z**	**P**
UVI Austria	Exploration-Avoidance	76.000	0.000	1.000
	Boldness-Shyness	55.000	-1.115	0.265
	Stress/Activity	72.000	-0.212	0.832
BBFS Brazil	Exploration-Avoidance	40.000	0.000	1.000
	Boldness-Shyness in Foraging	27.000	-1.155	0.248
	Boldness-Shyness in Predation	30.000	-0.889	0.374
	Sociability-Aggressiveness	**11.000**	**-2.577**	**0.010**
	Stress/Vigilance	37.000	-0.267	0.790

Table S11. PCA Personality Structure of Common Marmosets in Captivity (UVI Austria), in PTB1. Variable loadings in a principal component analysis (PCA), loadings >0.4 and <-0.4 were considered as salient, and high loadings >0.7 and <-0.7 are indicated in bold (Table is adapted from Table 1, Šlipogor et al. 2016).

**Component**	**Behavioral Variables**
Boldness-Shyness in Foraging	**(-0.930) Manipulation, tFUR**; **(-0.852) Proximity, mean**
	(+0.632) Distance, mean; **(+0.784) Vigilance calls, tFUR**; **(+0.890) Body latency, tFUR**; **(+0.934) Touch latency, tFUR**
Boldness-Shyness in Predation	**(-0.846) Body latency, tP**; (-0.616) Distance, mean; (-0.442) Vigilance calls, tP
	(+0.413) Proximity, mean; (+0.478) Stress behavior, tNF; **(+0.910) Contact calls, tP**
Stress-Activity	(-0.493) Touch latency, tNF; (-0.438) Manipulation, tNF
	**(+0.800) Locomotion, mean**; **(+0.804) Stress behavior, tNF**; **(+0.897) Compartment alternations, mean**
Exploration-Avoidance	(-0.616) Manipulation, tNF
	(+0.689) Touch latency, tNF; **(+0.794) Self-grooming, mean**; **(+0.824) Contact calls, GA**
